# Repeated Occurrence of Mobile Colistin Resistance Gene-Carrying Plasmids in Pathogenic *Escherichia coli* from German Pig Farms

**DOI:** 10.3390/microorganisms12040729

**Published:** 2024-04-03

**Authors:** Lisa Göpel, Ellen Prenger-Berninghoff, Silver A. Wolf, Torsten Semmler, Rolf Bauerfeind, Christa Ewers

**Affiliations:** 1 Institute of Hygiene and Infectious Diseases of Animals, Justus Liebig University Giessen, 35392 Giessen, Germany; lisa.goepel@vetmed.uni-giessen.de; 2 Department of Infectious Diseases and Microbiology, University of Luebeck, 23538 Luebeck, Germany; 3 Microbial Genomics, Robert Koch Institute, 13353 Berlin, Germany

**Keywords:** *Escherichia coli*, ETEC, EDEC, mobile colistin resistance, *mcr-1*, plasmid, IncX4, IncHI2, IncI2, swine

## Abstract

The global spread of plasmid-mediated mobile colistin resistance (*mcr*) genes threatens the vital role of colistin as a drug of last resort. We investigated whether the recurrent occurrence of specific *E. coli* pathotypes and plasmids in individual pig farms resulted from the continued presence or repeated reintroduction of distinct *E. coli* strains. *E. coli* isolates (*n* = 154) obtained from three pig farms with at least four consecutive years of *mcr* detection positive for virulence-associated genes (VAGs) predicting an intestinal pathogenic pathotype via polymerase chain reaction were analyzed. Detailed investigation of VAGs, antimicrobial resistance genes and plasmid Inc types was conducted using whole genome sequencing for 87 selected isolates. Sixty-one *E. coli* isolates harbored *mcr-1*, and one isolate carried *mcr-4*. On Farm 1, *mcr*-positive isolates were either edema disease *E. coli* (EDEC; 77.3%) or enterotoxigenic *E. coli* (ETEC; 22.7%). On Farm 2, all *mcr*-positive strains were ETEC, while *mcr*-positive isolates from Farm 3 showed a wider range of pathotypes. The *mcr-1.1* gene was located on IncHI2 (Farm 1), IncX4 (Farm 2) or IncX4 and IncI2 plasmids (Farm 3). These findings suggest that various pathogenic *E. coli* strains play an important role in maintaining plasmid-encoded colistin resistance genes in the pig environment over time.

## 1. Introduction

The emergence and spread of multidrug-resistant bacteria is a rising problem that threatens the effective treatment of infectious diseases in humans and animals [1]. *Escherichia* (*E*.) *coli* is a ubiquitous Gram-negative bacterium that is regularly found in the intestinal tract of humans and animals. Intestinal pathogenic *E. coli* (InPEC) strains can cause a number of different diseases, including diarrhea.

In pigs, neonatal and post-weaning diarrhea is a widespread and often severe disease, resulting in significant economic losses in the swine industry worldwide [2]. Certain *E. coli* pathotypes are associated with causing enteric diseases in piglets, e.g., enterotoxigenic *E. coli* (ETEC) and atypical enteropathogenic *E. coli* (aEPEC) [3]. *E. coli* isolates producing the Shiga toxin subtype Stx2e, encoded by the *stx2e* gene, are the causative agent of edema disease in weaned piglets [4]. Colistin has been widely used to treat intestinal infections in swine caused by InPEC [5]. In 2015, the use of colistin came under scrutiny due to the emergence of colistin-resistant bacteria due to a transferable colistin resistance gene [6]. A recent study from 2022 reported that 51.9% of 662 veterinarians surveyed stopped the use of colistin and 33.4% reduced their usage. The main indication for the use of colistin was gastrointestinal disease in pigs [6]. The increasing occurrence of antimicrobial resistance (AMR) against this last resort antibiotic incites new debates for additional regulations of its usage, especially in veterinary medicine [7].

Until a few years ago, acquired colistin resistance in bacteria was mainly attributed to chromosomal mutations such as modifications of two-component systems like *pmrA*/*pmrB* and *phoP*/*phoQ* [8,9]. In 2015, Liu et al. described for the first time a mobile colistin resistance gene that was found on a transmissible plasmid in one *E. coli* isolate from a Chinese pig, thereafter named *mcr-1* [10]. Since then, ten different *mcr* genes (*mcr-1*–*mcr-10*) have been reported with numerous variants, mostly found in Gram-negative bacterial species [11,12,13,14,15,16,17,18,19]. Over the years, *mcr*-mediated colistin resistance has been reported in Enterobacterales isolated from several sources, including humans, livestock, companion animals, wildlife and the environment [20,21,22,23]. 

The conjugative transfer of *mcr*-harboring plasmids between bacteria plays a vital role in the dissemination of AMR against colistin [24]. The first discovered *mcr-1* gene was located on a plasmid (pHNSHP45) of 64,015 bp in size, possessing a typical IncI2-type backbone [10]. Since then, *mcr-1* has been found on numerous plasmid types, such as IncHI1, IncHI2, IncF, IncN, IncP, IncX4 and IncN1-IncHI2 [25,26,27]. Resistance genes *mcr-2*, *mcr-3*, *mcr-6*, *mcr-7*, *mcr-8*, *mcr-9* and *mcr-10* were detected frequently on plasmids of types IncX4, IncHI2, IncHI2, IncI2, IncF type, IncHI2/HI2A and IncF, respectively [24].

The temporal and spatial distribution of *mcr*-positive isolates among pathogenic *E. coli* at individual farm levels has rarely been studied. Miguela-Villoldo et al. investigated *mcr-1* prevalence in healthy Spanish food-producing pigs from 1998 to 2021, selecting 50 caecal pig samples across 14 years [28]. While the frequency of *mcr-1*-positive samples increased from 2004 (16%) to 2015 (66%), a downward trend was observed from 2017 (54%) to 2021 (17%). In Germany, the *mcr-1* gene was detected in 15 different pig-fattening farms where pooled feces and boot swabs had been collected from 2011 to 2012 [29]. Further analyses of one representative *mcr-1*-containing *E. coli* isolate per farm showed that *mcr-1* was mainly located on IncX4 plasmids (*n* = 9) but also on IncHI2 (*n* = 3), IncX4/N (*n* = 1), IncHI2/FIB/FII/X3 (*n* = 1) and integrated into the chromosome (*n* = 1).

This study investigated the occurrence and genomic location of *mcr* genes in pathogenic *E. coli* isolates obtained from three German pig farms over at least four years, which were chosen from a comprehensive in-house database. The genomes of a representative set of isolates were sequenced to determine the presence of virulence-associated genes (VAGs) characteristic for intestinal pathogenic *E. coli* (InPEC) pathotypes and AMR genes as well as their plasmid location. Based on core genome and plasmid comparisons, the repeated occurrence of distinct *E. coli* clones, as well as distinct resistance and virulence plasmids on different farms, was examined. 

## 2. Materials and Methods

### 2.1. Study Inclusion Criteria for Farms

For this retrospective survey on the repeated occurrence of *mcr*-positive porcine pathogenic *E. coli*, suitable pig farms were selected based on specific criteria. From our database of more than 3000 registered pig farms in Germany that had sent samples for molecular typing of *E. coli* isolates in the past, we selected only farms from which at least 25 pathogenic *E. coli* isolates had been obtained and preserved over the years (*n* = 23 farms). This was based on our recently published collection of 10,573 *E. coli* isolates, each harboring at least one of ten VAGs, which were tested using PCR for the presence of *mcr-1* to *mcr-10* genes [30]. Briefly, *E. coli* isolates were obtained from feces or mucosal swabs (rectum or small intestine). Upon arrival at the laboratory, the samples were streaked for single bacterial colonies on blood agar plates (blood agar base, Merck Chemicals, Darmstadt, Germany) containing 5% sheep blood and on Gassner agar (sifin diagnostics GmbH, Berlin, Germany). After approximately 18 h of incubation at 37 °C, up to six morphologically different, putative *E. coli* colonies were picked per sample and tested for the presence of VAGs. More details of sample collection and processing were published recently [30].

Subsequently, pig farms with less than 25 pigs sampled (*n* = 11) or no detection of *mcr* genes (*n* = 5) were excluded from the study, as well as farms with no isolates for six consecutive years (*n* = 3). Finally, only farms were selected where *mcr*-positive *E. coli* had been isolated from pig fecal samples for at least four consecutive years.

### 2.2. Whole Genome Sequencing

The genomic DNA of *E. coli* bacteria was extracted using the Master Pure™ DNA Purification Kit (Biozym Scientific GmbH, Hessisch Oldendorf, Germany). Bacterial genomes were sequenced using an Illumina MiSeq sequencer (MiSeq Reagent Kit V.3; Illumina Inc., San Diego, CA, USA) via multiplexing of 30 samples per flow cell using 2 × 150 bp paired-end reads to achieve an average coverage of 90-fold. Quality control, including contamination removal and adapter trimming, was carried out using an in-house pipeline. De novo assemblies were generated using the SPAdes Genome Assembler (v3.15.5) with the “-isolate” flag [31]. The Bakta pipeline (v1.8.2) was employed using species-specific databases for genomic annotation of the bacterial genomes [32].

### 2.3. Phenotypic Resistance Testing, Antimicrobial Resistance Genes, Virulence-Associated Genes

All 87 whole genome-sequenced *E. coli* isolates were tested for antimicrobial susceptibilities using the broth microdilution method. An individual panel layout was used from the MICRONAUT system (Merlin Diagnostics, Bornheim-Hersel, Germany). Fourteen antimicrobial substances and/or combinations (in µg/mL: amikacin (0.25–32), amoxicillin/clavulanic acid (1/0.5–16/8), ampicillin (1–16), cefotaxime (0.016–2), ceftazidime (0.031–8), colistin (0.125–8), enrofloxacin (0.004–2), florfenicol (1–16), gentamicin (0.125–8), piperacillin/tazobactam (0.5/4–64/4), spectinomycin (4–64), sulfamethoxazole (4–256), tetracycline (0.25–8) and trimethoprim (0.25–8)) were included in this layout. *E. coli* strains ATCC 25922 and NCTC 13846 were used for quality control. Minimum inhibitory concentration (MIC) values were interpreted by means of defined clinical breakpoints set by the Clinical and Laboratory Standards Institute (CLSI) for veterinary and human Enterobacterales isolates [33]. For antibiotics without a defined breakpoint for *E. coli* (in the case of cefotaxime, sulfamethoxazole and trimethoprim), human breakpoints of Enterobacterales according to CLSI [34] were used, except for colistin, which was interpreted according to the European Committee on Antimicrobial Susceptibility Testing (EUCAST) guidelines [35]. No veterinary or human breakpoints are available for enrofloxacin and spectinomycin from either CLSI or EUCAST. For both substances, the epidemiological cut-off (ECOFF) was used for *E. coli* provided by EUCAST (https://mic.eucast.org/search/, accessed on 5 March 2024), separating the *E. coli* population into a population without acquired or mutational resistance (wild-type) and a population with phenotypically detectable acquired resistance mechanisms (non-wild-type) against enrofloxacin and spectinomycin. 

Antimicrobial resistance (AMR) genes and chromosomal point mutations related to antimicrobial resistance were identified in all whole genome-sequenced isolates by using the online tool ResFinder 4.1, available on the website of the Center for Genomic Epidemiology (CGE) (https://cge.food.dtu.dk/services/ResFinder/, accessed on 17 December 2023). All isolates were investigated for VAGs using VirulenceFinder 2.0 (https://cge.food.dtu.dk/services/VirulenceFinder/, accessed on 17 December 2023). The results were additionally verified with multiple genome analysis accessible online from BacWGSTdb (http://bacdb.cn/BacWGSTdb/Tools.php, accessed on 17 December 2023).

### 2.4. Determination of Genoserotypes, Clonotypes, Multilocus Sequence Types, Core Genome MLS Types and Phylogroups

The genoserotypes of the whole genome-sequenced *E. coli* isolates were determined by applying the web-based SerotypeFinder 2.0, provided by the Center for Genomic Epidemiology (https://cge.food.dtu.dk/services/SerotypeFinder/, accessed on 22 November 2023). The internal 469- and 489-nucleotide sequences of the *fumC* and *fimH* genes, respectively, were used for clonotyping [36]. Clonotypes (CH types), which are the combinations of allele assignments for *fumC* and *fimH*, were determined using CHTyper 1.0 (https://cge.food.dtu.dk/services/CHTyper/, accessed on 1 January 2024).

Multilocus sequence types (STs) were determined by applying MLST 2.0 (https://cge.food.dtu.dk/services/MLST/, accessed on 22 November 2023), which is based on the Achtman seven-gene MLST scheme that includes seven housekeeping genes (*adk*, *fumC*, *gyrB*, *icd*, *mdh*, *purA* and *recA*). *E. coli* isolates were defined as clonal when they displayed the same sequence type in addition to identical virulence and resistance gene profiles.

The core genome was calculated using Roary [37]. Phylogenetic analysis was performed by using the web-based tool ClermontTyper (IAME, http://clermontyping.iame-research.center/index.php, accessed 18 December 2023), allowing us to assign tested strains to *E. albertii*, *E. fergusonii*, *Escherichia* cryptic clades I-V, *E. coli sensu strico* as well as to the main phylogroups A, B1, B2, C, D, E, F and G [38,39]. The remaining non-sequenced *E. coli* isolates were tested in a quadruplex PCR, allowing for the assignment to the eight main phylogroups A, B1, B2, C, D, E, F, G and *Escherichia* cryptic clades I-V [39,40].

### 2.5. Genomic Location of Virulence-Associated Genes and mcr Genes, Plasmid Analysis

To determine the location of *mcr* genes, two methods were used. Whole genome sequence data were analyzed for the location of AMR and virulence genes using the tool “Chromosome & Plasmid Overview” implemented in the software package Ridom SeqSphere_+_ (http://www3.ridom.de/seqsphere, accessed 22 January 2024). To identify plasmid replicon types, PlasmidFinder 2.1 (https://cge.food.dtu.dk/services/PlasmidFinder/, accessed 22 January 2024) and Ridom SeqSphere+ were employed. The BacWGST database (http://bacdb.cn/BacWGSTdb/Tools.php, accessed on 17 December 2023) and RidomSeqSphere+ were applied to detect closely related plasmids via sequence comparison. To illustrate circular comparisons between the plasmids, we used the blast ring image generator software BRIG Version 0.95 [41].

To confirm the results of sequence analysis regarding the genomic location of *mcr*-*1* genes, an additional method was employed. The location of *mcr-1* genes in 38 *E. coli* isolates was determined via the S1 nuclease digestion of genomic DNA and pulsed-field gel electrophoresis (PFGE), followed by Southern blot hybridization (SBH). For this method, digoxigenin-labeled DNA probes (“DIG luminescent detection Kit” Boehringer Mannheim GmbH, Mannheim, Germany) were generated, targeting the PCR fragments specific for the *mcr-1* gene [10].

## 3. Results

### 3.1. Farm Selection

Farms in Hesse (*n* = 2) and North Rhine Westphalia (*n* = 1), Germany, met the chosen study inclusion criteria. Farm 1 sent samples (41 × feces, 1 × intestine + feces, and 1 × *E. coli* isolate) from May 2002 to October 2019. Samples were obtained from 43 pigs, most of them suffering from diarrheal disease. From this sample material, 50 *E. coli* isolates were cultivated that were positive for at least one VAG associated with certain pathotypes of InPEC as determined by PCR [30]. Twenty-two (44%) of these *E. coli* isolates, obtained from July 2009 to September 2012, tested positive for *mcr-1* genes (Table 1).

Farm 2 submitted samples from 69 pigs between June 2004 and February 2021. Samples were mainly feces (*n* = 43), followed by directly submitted *E. coli* isolates (*n* = 22) and by isolates obtained from the intestine and feces (*n* = 4). From these samples, 78 VAG-positive *E. coli* isolates were stored. Twenty-four (30.8%) isolates, obtained from July 2013 to February 2018, proved *mcr-1*-positive (Table 1).

From October 2014 to April 2019, 26 VAG-positive *E. coli* were isolated from 25 sampled pigs of Farm 3. Most isolates (*n* = 23) were received through submissions from other veterinary diagnostic laboratories for further molecular typing in our institute, supplemented by two fecal samples. In total, 16 (61.5%) *mcr*-positive pathogenic *E. coli* were retrieved over more than four years (Table 1).

In total, 154 intestinal pathogenic *E. coli* isolates were obtained from 137 pigs housed on the selected three farms from May 2002 to February 2021. The total number of *mcr*-positive isolates was 62 (40.3%).

### 3.2. E. coli Pathotypes

A prediction of pathotypes was conducted for all 154 *E. coli* isolates included in this study based on the presence of certain VAGs obtained from PCR analysis, as described previously [30]: adhesive fimbriae *E. coli* (in the following termed AdhF-*Ec*), positive only for at least one adhesive fimbriae gene (*faeG, fanA, fasA, fedA* and *fimF41a*); AEEC (often also referred to as atypical EPEC), positive for *eae*; EDEC, positive for *fedA* and *stx2*; ETEC, positive for at least one adhesive fimbriae gene (*faeG, fanA, fasA, fedA* and *fimF41a*) and at least one enterotoxin gene (*eltB-Ip, estap* and *estb*); ETEC-like, positive only for at least one enterotoxin gene (*eltB-Ip, estap* and *estb*); ETEC/STEC hybrid (in the following simply termed ETEC/STEC), positive for at least one adhesive fimbriae gene (*faeG, fanA, fasA, fedA* and *fimF41a*) and at least one enterotoxin gene (*eltB-Ip, estap* and *estb*) and *stx2*; STEC, positive only for *stx2*.

The most common pathotypes detected in all farms were ETEC (*n* = 78; 50.7%) and EDEC (*n* = 32; 20.8%). ETEC/STEC (*n* = 16; 10.4%) and ETEC-like (*n* = 12; 7.8%) were found in farms 2 and 3 and all three farms, respectively. AEEC (*n* = 6; 3.9%), STEC (*n* = 6; 3.9%) and AdhF-*Ec* (*n* = 4; 2.6%) were rarely identified. Details about the distribution of *E. coli* pathotypes according to farms are given in Table 1. 

Additionally, with regard to *mcr-1*-positive pathogenic *E. coli*, ETEC (*n* = 29; 48.3%) and EDEC (*n* = 18; 30%) were the most common pathotypes, followed by ETEC/STEC (*n* = 11; 18.3%) and STEC (*n* = 2; 3.3%). On Farm 1, 81% of the *mcr-1*-positive isolates were EDEC (*n* = 17) and 19% ETEC (*n* = 4) (Table 1). On Farm 2, all *mcr-1*-positive isolates were ETEC. The distribution of *mcr-1*-positive isolates on Farm 3 was as follows: ETEC/STEC (*n* = 11; 73.3%), followed by EDEC (*n* = 2; 13.3%), STEC (*n* = 1; 6.7%), and ETEC (*n* = 1; 6.7%).

### 3.3. Virulence Associated Genes and Virulence Plasmids

The genomes of 87 *E. coli* isolates were sequenced to conduct further molecular analysis, specifically regarding VAGs, as well as *mcr* detection and genomic localization. The isolates included 38 *mcr-1.1*-positive, 1 *mcr-4.8*-positive and 48 *mcr*-negative *E. coli* isolates (Table 2). At least one isolate per year and farm was selected for genome sequencing. If there were several *mcr*-positive isolates of different pathotypes per year and farm, at least one representative isolate per pathotype was selected. The *mcr*-negative isolates (one per pathotype) were additionally sequenced from the years with detected *mcr* occurrence. 

The majority of the VAGs identified via PCR (Section 3.1) were also identified in the genomic data (Table 2). The adhesive fimbriae F18 (encoded by the *fedA* gene) were further classified into F18 fimbrial subtypes F18ab (*fedAab*) and F18ac (*fedAac*). In total, 19 isolates encoded for the F18ab subtype, while 21 isolates encoded for F18ac. One isolate carrying the *fedA* gene was not further typeable (Farm 1, IHIT52950). EDEC, defined as isolates positive for *fedA* and *stx2e*, was predicted in 94.7% of all F18ab-positive bacteria. Isolates encoding for F18ac mostly harbored enterotoxin genes (ETEC; 61.9%) or enterotoxin and Shiga toxin genes (ETEC/STEC; 33.3%). All of the isolates that tested positive for F4 fimbriae (encoded by *faeG*) in the PCR were identified as the F4ac subtype (*faeGac*) via genome analysis. This subtype is known to be the most prevalent in piglets with post-weaning diarrhea [2]. Genomic data from 30 isolates positive for the *stx2* gene via PCR revealed that they encoded for Shiga toxin subtype Stx2e (*stx2e*), which plays a pivotal role in the pathogenesis of edema disease in swine [4].

**Figure 1:**
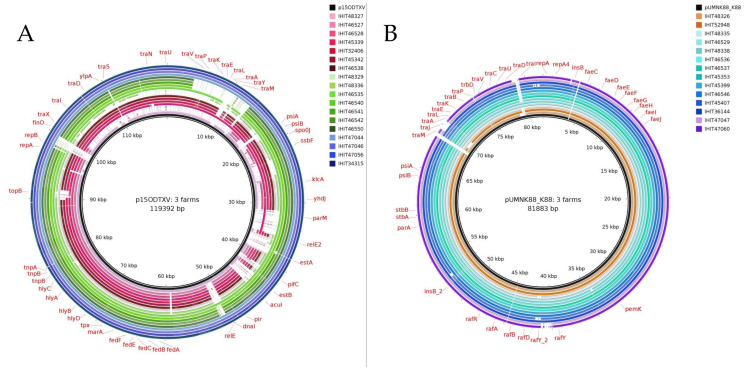
Schematic circular representation of *E. coli* virulence plasmids detected in all three farms over the years (details given in Table 2). For comparison, one representative isolate per year and farm was selected. The reference plasmids used were p15ODTXV (**A**); NCBI Reference Sequence: MG904998.1) and pUMNK88_K88 (**B**); NCBI Reference Sequence: CP002730.1), shown in the inner rings. The plasmids detected in isolates from the three farms are arranged according to farm and year of isolation. Isolates from the same farm are color-coded (**A**): Farm 1 = red, Farm 2 = green, Farm 3 = blue; (**B**): Farm 1 = orange, Farm 2 = turquoise/blue, Farm 3 = violet). The color of the earliest isolation is displayed as the lightest, while the color of the latest isolation is displayed as the darkest.

Over the years, *E. coli* isolates from all three farms repeatedly harbored similar virulence plasmids that either carried fimbrial genes, enterotoxin genes or both. Two reference plasmids were identified in the NCBI database, which showed high similarities to these virulence plasmids. To illustrate similarities between the study and reference plasmids, we selected one representative isolate per year and farm for BRIG analysis (Figure 1). Table 2 provides details on the *E. coli* strains used as representative isolates, including isolation year, pathotype, and ST.

Reference plasmid p15ODTXV (NCBI Reference Sequence: MG904998.1) was identified in an ETEC/STEC strain isolated from a diarrheic pig in Switzerland in 2014/2015. It carried *estap*, *estb*, *fedA* and *hlyDBAC* virulence genes. Representative plasmids identified in four isolates from Farm 3 were highly similar to the IncFII/IncX1 multivirulence reference plasmid used in Figure 1A. Virulence plasmids detected in Farm 1 (*n* = 19) and Farm 2 (*n* = 10) resembling p15ODTXV showed varying similarities over the years and mostly carried either *fedAab* (*n* = 15) or *fedAac* (*n* = 11; Table S1) genes.

Plasmids with high similarity to the IncFII reference plasmid pUMNK88_K88 (NCBI Reference Sequence: CP002730.1) were found in all three farms. In 2007, one ETEC strain from a pig with neonatal diarrhea in the USA was found to contain the pUMNK88_K88 plasmid, which carried *faeG* as a virulence gene. BLAST analysis revealed two very similar IncFII plasmids harbored by two strains positive for *faeGac* from Farm 1 and Farm 3, respectively (Table S1). In Farm 2, IncFII plasmids encoding for *faeGac* were found in ten ETEC strains (all ST100) isolated in the years 2005 to 2018 (Figure 1B). A comparison of the plasmids from this study with reference plasmids demonstrated structural resemblance of virulence plasmids over several years within and, in part, also across farms (e.g., plasmid pUMNK88_K88).

Table S2 provides a detailed distribution of all additionally detected VAGs among whole genome-sequenced isolates, sorted by farms and the categories: adhesion, tissue damage (hemolysin/toxin genes), invasion and protection, iron acquisition and secretion system.

### 3.4. Resistance Phenotypes and Genotypes

Most of the 87 isolates were resistant to ampicillin (86.2%; >16 µg/mL), sulfamethoxazole (88.5%; >256 µg/mL) and tetracycline (86.2%; >8 µg/mL). Lower resistance rates were observed with trimethoprim (42.5%; ≥16 µg/mL), gentamicin (9.2%; >8 µg/mL), florfenicol (4.6%; >16 µg/mL) and cefotaxime (1.1%; >2 µg/mL). Low MIC values were determined for enrofloxacin (83.9%; <0.125 µg/mL), assigning most of the isolates to a wild-type population, as defined by ECOFFs provided by EUCAST. More than half of the tested strains showed MIC values over 64 µg/mL for spectinomycin (62.1%), categorizing these isolates into a non-wild-type population. No isolate was resistant to amoxicillin/clavulanic acid (≥32/16 µg/mL), amikacin (≥64 µg/mL), ceftazidime (≥16 µg/mL) and piperacillin/tazobactam (≥128/4 µg/mL).

Colistin MICs ranged from 0.25 to 8 µg/mL. Fourty-one isolates proved susceptible to colistin, while fourty-six strains were resistant (52.9%; ≥4 µg/mL). Almost all *mcr*-positive isolates showed resistance to colistin, with a MIC ≥ 4 µg/mL, except for IHIT25408 (Farm 2) and IHIT47044 (Farm 3), which showed MICs of 0.5 µg/mL. The chromosomal point mutation in the *pmrB* gene (V161G), which is presumably associated with colistin resistance, was detected in four *mcr*-negative isolates (Farm 1: 2 ST42-ETEC; Farm 2: 1 ST42-AdhF-*Ec*; 1 ST42-ETEC). Two of these isolates showed phenotypic resistance to colistin (Table 2).

The same AMR genes were prevalent in all three farms, with *sul2* (85.2%), *aph(3``)-Ib* (77.8%), *aph(6)-Id* (77.8%), *bla*_TEM-1B_ (77.8%) and *tet(A)* (74.1%) being the most frequently detected AMR genes in Farm 1. In Farm 2, the most prevalent AMR genes detected were *tet(A)* (79.6%), *bla*_TEM-1B_ (72.7%), *sul2* (68.2%), *aadA1* (45.5%), *aph(3``)-Ib* (43.2%) and *aph(6)-Id* (43.2%). In Farm 3, the AMR genes most frequently found were *dfrA1* (75%), followed by *aph(3``)-Ib* (68.8%), *aph(6)-Id* (68.8%), *bla*_TEM-1B_ (68.8%), *sul1* (68.8%), *sul2* (68.8%), *aadA1* (62.5) and *tet(A)* (62.5%). Chromosomal mutations in the *gyrA* and *parE* genes were identified in all three farms. The highest prevalence was observed in Farm 3, with 50% of the isolates showing the *gyrA* S83L mutation and 43.8% showing the *parE* I355T mutation. Details on the distribution of AMR genes and chromosomal point mutations are provided in Table S3. 

AMR gene patterns (excluding *mcr* genes) differed among the *E. coli* isolates over time. In Farm 1, ST1-EDEC isolates from December 2005 to October 2019 revealed five different combinations of AMR genes (Table S3). In Farm 2, ST100-ETEC strains exhibited mainly two different AMR gene patterns over the years. Between November 2011 and May 2014, ST100-ETEC strains were found to carry multiple AMR genes, including *aadA5, aph(3``)-Ib, aph(6)-Id, bla*_TEM-1B_*, catB3, dfrA1, dfrA14, sul1, sul2* and *tet(A)*. However, ST100-ETEC strains isolated from October 2014 to February 2018 only tested positive for the genes *bla*_TEM-1B_*, sul2* and *tet(A)*. On Farm 3, all isolates obtained over the time revealed different AMR gene patterns.

### 3.5. Multilocus Sequence Types, Clonotypes, Phylogenetic Groups

**Figure 2:**
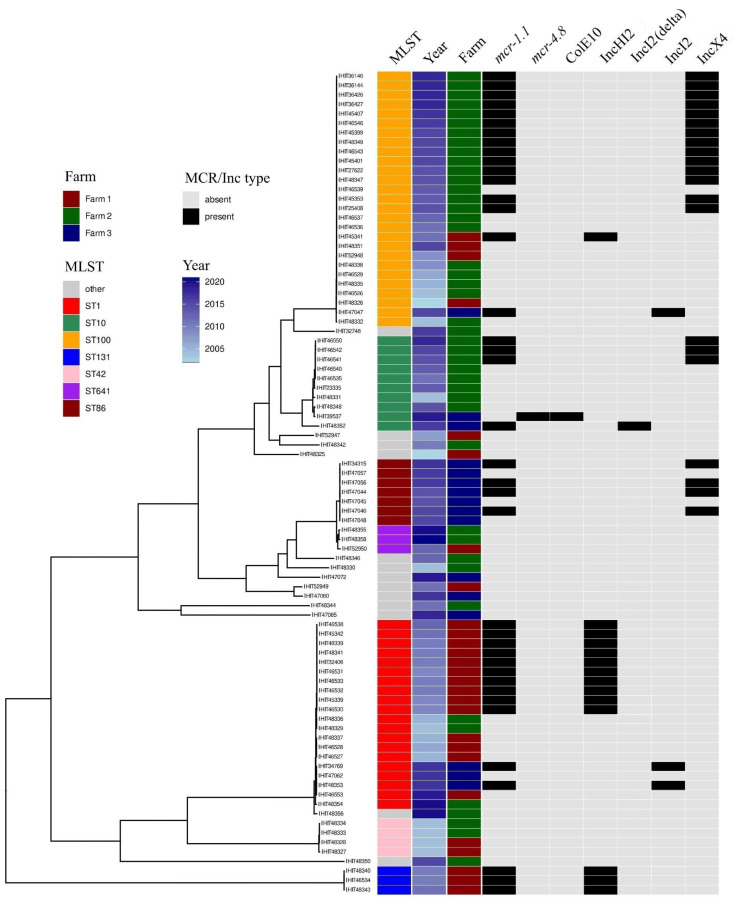
Neighbor-joining tree based on the comparison of 2670 core genome genes of 87 *E. coli* isolates from Farm 1, Farm 2 and Farm 3, with the respective multilocus sequence types (MLST) and isolation years color-coded. The occurrence of *mcr* genes and their location on different plasmids are shown. Farm specific clustering of isolates from different years can be observed for Farm 1, Farm 2 and Farm 3.

In the phylogenetic tree of all whole genome-sequenced *E. coli* isolates in this study, the majority of isolates from each farm clustered together, irrespective of the year of isolation (Figure 2). Clustering was also observed for *mcr*-positive isolates on each farm.

Overall, 20 known multilocus sequence types (STs) were determined (Table 2). ST1 was the predominant ST (60.9% of the isolates) on Farm 1, while the most prevalent STs on farms 2 and 3 were ST100 (*n* = 21; 48.8%) and ST86 (*n* = 7; 43.8%), respectively (Figure 2). Three *mcr-1*-positive ETEC isolates from Farm 2 (isolated between June 2010 and June 2011) belonged to ST131. This sequence type has previously been detected in ETEC strains isolated from pigs with diarrhea in Spain [3]. It is also known as the predominant *E. coli* lineage among extraintestinal pathogenic *E. coli* (ExPEC) worldwide, frequently associated with an ESBL phenotype and multidrug resistance [42]. 

Nine, fifteen and eight different clonotypes (CH types) were found in *E. coli* isolates from farms 1, 2 and 3, respectively (Table 2). The most frequent CH types on Farm 1 and Farm 2 were 2-54 (51.9%; all ST1-EDEC) and 27-0 (50%; all ST100-ETEC), respectively. CH type 6–32 was most common in Farm 3 (43.8%, 7 ST86-ETEC/STEC and one ST86-ETEC).

**Figure 3:**
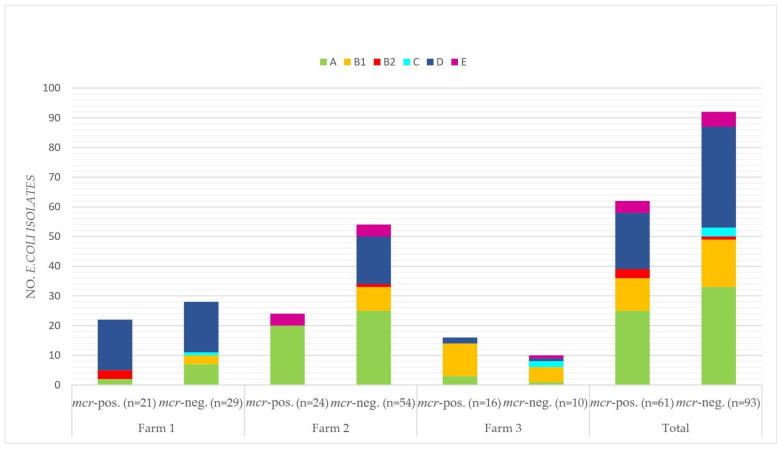
Overview of the phylogenetic grouping of *mcr*-positive and *mcr*-negative *E. coli* isolates sorted by Farms 1 to 3 and in total.

Phylogroups A (*n* = 58; 37.7%) and D (*n* = 53; 34.4%) were found to be the most common phylogroups among all 154 *E. coli* isolates (Figure 3). While the majority of *E. coli* isolates of Farm 1 were assigned to phylogroup D (*n* = 34; 68%), phylogroups A (*n* = 45; 57.7%) and B1 (*n* = 16; 61.5%), which were most frequent in isolates from Farms 2 and 3, respectively. Overall, only four, three and nine isolates were assigned to phylogenetic groups B2, C and E. No isolate belonged to phylogroup F or G. 

Considering only isolates that were positive for *mcr* genes, a similar distribution of phylogroups per farm and in total was observed. The predominant phylogroups for farms 1 to 3 were D (*n* = 17; 81%), A (*n* = 20; 83.3%) and B1 (*n* = 11; 68.8%), respectively. Only phylogroups A and E (*n* = 4; 16.7%) were determined for *mcr*-positive isolates of Farm 2. No *mcr*-positive *E. coli* belonged to phylogroup C.

### 3.6. Genoserotypes

A total of 27 different genoserotypes were identified among 87 whole genome-sequenced *E. coli* isolates (Table 2). Fourteen isolates were not typeable (Ont). The most common genoserotype in isolates obtained from Farm 1 was O139:H1 (*n* = 13; 56.5%). This genoserotype has been associated with edema disease-causing *E. coli* in pigs in Europe [43]. Genoserotype O149:H10 was found repeatedly in isolates from Farm 2 (*n* = 18; 41.9%). Commonly reported O antigens of ETEC and EDEC isolates, such as O45, O139, O141, O147 and O149, were predominant (*n* = 52; 71.2%) among all typeable isolates of this study.

### 3.7. Genomic Location of mcr Genes and Plasmid Analysis

The genomic location of *mcr-1.1* in 38 whole genome-sequenced *E. coli* isolates was determined via Southern blot hybridization (SBH) of S1 nuclease-digested whole-cell DNA and analysis of genomic sequence data. We identified *mcr-1.1* on four different plasmids in this study. S1 nuclease PFGE and SBH initially revealed *mcr* genes on plasmids with approximate sizes of 250 kbp (*n* = 13), 35 kbp (*n* = 19) and 65 kbp (*n* = 3). Three isolates (IHIT34315, IHIT34769, and IHIT36427) did not reveal the genomic location of *mcr* genes through SBH despite repeated attempts. 

Additionally, whole genome sequence analysis confirmed the occurrence of identical *mcr-1.1*-harboring plasmids per farm. All of the tested *mcr*-positive *E. coli* of Farm 1 carried *mcr-1.1* on IncHI2 plasmids (*n* = 14), while all 17 *mcr*-positive isolates of Farm 2 harbored *mcr-1.1* on IncX4 plasmids. *E. coli* isolates obtained from Farm 3 carried the *mcr-1.1* gene on IncX4 (*n* = 4), IncI2 (*n* = 3) and IncI2 (delta) (*n* = 1) plasmids. The *mcr-4.8* gene was located on a ColE10 plasmid, as previously reported [30].

**Figure 4:**
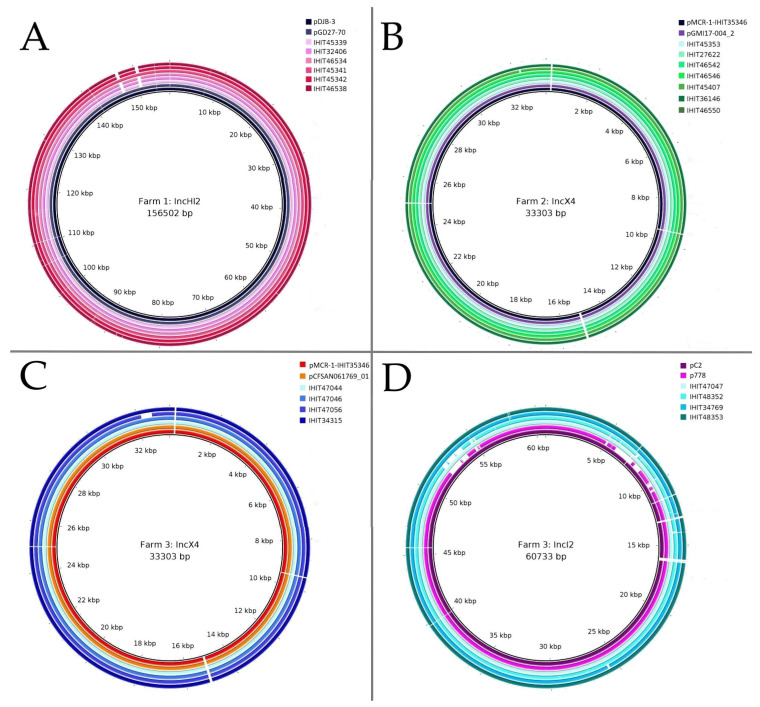
Schematic circular representation of detected MCR-1 plasmids from three farms in our study compared to most similar reference plasmids predicted using the BacWGST database. One representative *mcr*-positive *E. coli* isolate per year and farm was selected for comparison (details given in Table 2). If two different *mcr*-positive pathotypes were detected per year and farm, we selected both pathotypes. The plasmids from our study are arranged in order of isolation year, with the earliest (light color) on the third innermost ring and the latest (dark color) on the outermost ring in (**A**–**D**). The reference plasmids are shown in the two innermost rings. Reference plasmids (NCBI Reference Sequence in brackets) included are as follows: pDJB-3 (MK574666.1), pGD27-70 (MN232195.1) for Farm 1 (**A**); pMCR-1-IHIT35346 (KX894453.1), pGMI17-004_2 (NZ_CP028167.1) for Farm 2 (**B**); pMCR-1-IHIT35346 (KX894453.1), pCFSAN061769_01 (CP042970.1) for Farm 3 (**C**); pC2 (LC473131.1) and p778 (MN746292.1) for Farm 3 (**D**).

The BacWGST database was used to identify closely related MCR-1 plasmids, which were then used as reference plasmids to illustrate circular comparisons with MCR-1 plasmids of representative isolates of this study (Figure 4).

For comparison, we selected one *mcr*-positive representative *E. coli* isolate per year and farm. When two different pathotypes tested positive for *mcr* in the same year, both positive pathotypes were included. Six representative plasmids were detected in Farm 1 and identified as similar to two IncHI2 reference plasmids. The plasmids were found in IHIT45339 (EDEC; 2009), IHIT32406 (EDEC; 2010), IHIT46534 (ETEC; 2010), IHIT45341 (ETEC; 2011), IHIT45342 (EDEC; 2011) and IHIT46538 (EDEC; 2012), with nucleotide sequence identities ranging from 98.8% to 99.99% and coverage ranging from 90% to 97% compared to the references. The reference plasmids harboring *mcr-1.1* had been identified in *E. coli* strains isolated from swine in China (pDJB-3; NCBI Reference Sequence: MK574666.1; used as a reference plasmid in Figure 4A) and broiler chicken in China (pGD27-70; NCBI Reference Sequence: MN232195.1). 

The reference plasmid used for *mcr*-harboring plasmids from ETEC isolates in Farm 2 was the *mcr-1.1*-carrying IncX4 plasmid identified in an *E. coli* isolate obtained from pig feces in Italy in 2016 (pMCR-1-IHIT35346; identity ≥ 99.4%, coverage ≥ 95%; NCBI Reference Sequence: KX894453.1). Another very similar *mcr-1.1*-carrying IncX4 plasmid (pGMI17-004_2; NCBI Reference Sequence: NZ_CP028167.1) obtained from an *E. coli* isolate from poultry in Denmark in 2010 is also represented in Figure 4B.

The IncX4 plasmid pMCR-1-IHIT35346 was also used as a reference for all IncX4 plasmids detected in Farm 3 (identity ranging from 97.4% to 100%, coverage ≥ 98%) (Figure 4C). Additionally, one *E. coli* strain, isolated from raw milk cheese in Egypt in 2016, harbored one *mcr-1.1*-carrying IncX4, which showed high identity (≥ 99.96%, coverage ≥ 98%) to all four IncX4 plasmids from Farm 3 (pCFSAN061769_01; NCBI Reference Sequence: CP042970.1).

An *E. coli* isolate obtained from influent municipal wastewater in Japan harbored *mcr-1.1*-carrying IncI2 plasmid pC2 (NCBI Reference Sequence: LC473131.1), which was used as a reference in Figure 4D. This plasmid was highly similar to plasmids from Farm 3 (isolates IHIT47047, IHIT34769, IHIT48353), with identities ranging from 92.66% to 99.99% and coverage greater than 99%. However, IHIT48352 harbored one IncI2 (delta), which was similar to one *mcr-1.1*-carrying plasmid from a *Salmonella enterica* isolate obtained from a child in Ecuador (p778; 98.4% identity, coverage 100%; NCBI Reference Sequence: MN746292.1). None of the selected reference plasmids contained additional resistance genes besides *mcr-1.1*. 

## 4. Discussion

The global dissemination of colistin resistance genes in bacteria from humans, animals and the environment has been reported over the last years [24,44]. The occurrence of *mcr* genes has been investigated in various studies, especially those conducted concerning pig and poultry production [45,46,47]. However, data on recurrent *mcr*-mediated resistance in livestock farms are scarce [48,49]. We were not able to identify clones of pathogenic *E. coli* over the years on the specific farms. However, our study described three German pig farms with the repeated occurrence of highly similar *mcr*-carrying plasmids in pathogenic *E. coli* isolates over two to more than four years, respectively. 

In our study, ETEC was the prevalent pathotype for all collected isolates (78/154; 50.7%) as well as for *mcr-1*-positive isolates (30/61; 49.2%). The most common pathotypes for each farm also coincided with the prevalent pathotypes of *mcr-1*-harboring *E.coli*. ETEC has been reported to be the most common cause of post-weaning diarrhea (PWD) in swine, but is also known as a pathogen in humans [5,50]. An epidemiological study from Spain reported a significant association of ETEC (67%) with the occurrence of PWD in swine [3]. That study investigated 481 *E. coli* isolates from diarrheic pigs, of which 123 (25.6%) strains carried the *mcr-1* gene, with 57.7% belonging to the ETEC pathotype. This study also investigated the prevalence of the Shiga toxin gene *stx2*, which was found to be lower than in our study (10% vs. 35%) [3].

We identified IncFII/IncX1 and IncFII plasmids as the most frequent virulence plasmid types among the isolates from all three farms over the years. VAGs like *eltB-Ip*, *estap*, *estb*, and *fedABCEF* have previously been reported to be encoded on IncF or IncFII/IncX1 plasmids of *E. coli* strains isolated from diarrheic pigs [51,52]. Virulence plasmids have occasionally been described in ETEC and ETEC/STEC strains [52,53], but to the best of our knowledge, not in individual pig farms over the years. The striking similarity of certain virulence plasmids in single farms, such as in Farm 3 for four consecutive years (Figure 1A), suggests a local clonal distribution of virulence plasmids. However, it should be taken into account that specific virulence plasmids can be distributed across farms (Figure 1B) and countries. In summary, our data do not support clones (according to the strict definition used in our study design) of *E. coli* strains carrying specific virulence plasmids over the years.

Effelsberg et al. investigated the occurrence of *mcr-1* to *mcr-5* genes in 318 porcine fecal samples from 81 pig farms in northwest Germany collected from March 2018 to September 2020 [54]. Overall, ten farms (12.3%) provided fecal samples that contained *E. coli* harboring *mcr-1*. Two farms showed repeated presence of *mcr-1*-positive strains from two different dates of sampling. A retrospective study examined 436 boot swab and pooled fecal samples from 58 German pig-fattening farms for the presence of *mcr-1* and *mcr-2* [29]. The *mcr-1* gene was detected in 43 (9.9%) *E. coli* isolates obtained from 15 (25.9%) farms, which were almost evenly distributed in northern/western (25%), southern (25%), middle (21%) and eastern (36%) Germany in 2011 to 2012. Considering the presence of *mcr*-positive pathogenic *E. coli* strains on the farms examined in our study, the prevalence was high, with 42%, 30.8% and 61.5% for farms 1, 2 and 3, respectively. A longitudinal study from Thailand investigated the occurrence of *mcr* genes in one representative pig farm from 2017 to 2020 after the cessation of prophylactic colistin usage [48]. Samples were taken from pigs (*n* = 70), farm workers (*n* = 50) and wastewater (*n* = 50). While *mcr-1*-positive isolates (*n* = 4; 8%) were detected in humans only in 2017, the prevalence of colistin-resistant strains was 28.6% in pigs, with a declining trend over the years, and 18% in wastewater. Another study obtained mixed bacterial cultures (*n* = 35) from the environment of three German pig farms from 2011 to 2012 [55]. Seven *E. coli* isolates tested positive for *mcr-1*-harboring IncX4 plasmids. The positive samples were obtained from boot swabs, barn dog feces, stable flies and manure. In our study, we did not expect the recurrent identification of highly similar *mcr*-positive pathogenic *E. coli* isolates in three German pig farms over more than four years. This is a concerning observation because it suggests that the occurrence of resistant strains over the years may be largely undetected due to missing systematic observational data.

The predominant *mcr-1.1*-harboring plasmid types in our study were IncHI2 for farm 1, IncX4 for Farm 2 and both IncX4 and IncI2 plasmids for Farm 3. A systematic review by Matamoros et al. reported a total of 13 plasmid incompatibility types for *mcr-1*-carrying plasmids for 217 *Enterobacteriaceae* isolated from human, animal and environmental samples [56]. The majority of plasmid types were IncX4 (35.2%), IncI2 (34.7%) and IncHI2 (20.5%), with a significant geographical clustering of IncHI2 plasmids in Europe and a regional spread of IncI2 plasmids in Asia. A recent study from France investigated the occurrence of *mcr-1*–*mcr-5* and *mcr-9* genes in colistin-resistant *E. coli* isolates obtained from over 1500 goats of 80 breeding and five fattening goat farms [57]. In total, 149 *mcr-1*-positive *E. coli* were identified, with 146 *mcr-1* genes located on either IncX4 (38.9%) or IncHI2 (26.8%) plasmids and on the chromosome (32.2%). The *mcr-1*-carrying plasmids of types IncX4 and IncHI2 were never detected on the same farm in that study.

The most frequently detected sequence types in our study were ST100 (*n* = 27; all ETEC) and ST1 (*n* = 20; 19 EDEC, 1 AdhF-*Ec*). Kusumoto et al. (2016) investigated 967 swine-pathogenic *E. coli* strains that were isolated from diseased pigs in Japan between 1991 and 2014 [58]. Isolates that were classified as EDEC in that study predominantly belonged to ST1, while ETEC strains were mostly typed as ST100. Sequence type ST10, which was the third most common sequence type in our study (*n* = 10, 50% *mcr*-positive isolates), was the most prevalent ST in *mcr*-positive swine-pathogenic *E. coli* in a study from Spain [3]. Three *mcr-1.1*-positive ST131 ETEC strains (harboring ETEC-typical genes *fedAac, estap* and *estb*) were isolated from Farm 2 in June 2010 (*n* =2) and June 2011 (*n* = 1). All strains harbored additional virulence genes *fimH*, *fyuA*, *hlyA*, *ibeA*, *iss*, *kpsMII*, *ompA*, *papB*, *papC*, *sitA* and *traT*, which are typical for ExPEC [59].

Bok et al. analyzed 274 *E. coli* strains isolated from healthy post-weaning piglets and sows in Poland with the phylogenetic assignment of isolates mainly to phylogroups A (37.6%) and B1 (33.2%) [60]. This is in line with our findings of phylogroups A and B1 being the most prevalent phylogroups for Farm 2 and Farm 3, respectively. Another study from Thailand reported phylogroups A (44.3%), B1 (34.4%) and D (14.8%) being the predominant phylogroups for *mcr*-harboring *E. coli* from slaughtered pigs in 2014–2015 [61]. Here, we observed similar prevalences for collected *mcr*-positive *E. coli* isolates.

Our study has some limitations. This retrospective survey was based on the receipt of samples for diagnostic purposes and allows for bias due to the random nature of sample submissions. Only porcine *E. coli* isolates that proved positive for certain VAGs were documented and stored, leaving out non-pathogenic *E. coli*. In addition, viable but non-culturable (VBNC) *E. coli* were not studied. Bacteria in the VBNC state are viable but unable to grow on nutrient culture media. Pathogenic *E. coli* entering the VBNC state as a survival strategy has been reported [62], and would have been missed in our study design. Furthermore, comprehensive data were not available for all three farms, including information on farm characteristics such as potential external contamination, management practices related to animal health, and previous veterinary treatments, including the use of colistin or other antimicrobials.

The collection of a large pool of pathogenic *E. coli* isolates over a period of more than twenty years is a major advantage for the selection and evaluation of sampled pig farms in Germany. To the best of our knowledge, this study is the first to present phenotypic and molecular data on *mcr*-mediated resistance and intestinal pathogenic *E. coli* isolates from individual pig farms over an extended period of time.

## 5. Conclusions

We describe three German pig farms in which pathotypes of *E. coli*, including ETEC and EDEC, and various MCR plasmids have been found repeatedly over the years. Isolates obtained on each farm over 17.4, 26.6, and 4.5 years, respectively, differed from each other in their multilocus sequence types and/or VAG and AMR gene profiles and were therefore not considered clonally related. However, a comparison of the plasmid sequence data with reference plasmids demonstrated the structural resemblance of virulence plasmids over several years within and, in part, also across farms.

The data suggest that the repeated occurrence of *mcr*-carrying plasmids in pathogenic *E. coli* isolates may be due to the local long-term occurrence of *mcr*-carrying plasmids rather than reinfection with different plasmids. In affected farms, specific eradication programs, such as rigorous hygiene management and prevention of pig trafficking, may be at least as equally important and effective in reducing the burden of colistin resistance as general recommendations to reduce colistin usage.

## Figures and Tables

**Table 1:** Sample collection from three farms and predicted pathotypes from all *E. coli* isolates and *mcr*-positive *E. coli* isolates per farm.

			*E. coli* Isolates Possessing InPEC-Related VAGs *			*mcr*-Positive *E. coli* Isolates Possessing InPEC-Related VAGs		
Farm No.	No. of Sampled Pigs	Sample Type (No.)	No.	Predicted Pathotypes **	Sample Collection Period	No.	Predicted Pathotypes	Isolate Collection Period
Farm 1	43	feces (41), intestine + feces (1), isolate (1)	50	EDEC (23), ETEC (21), STEC (3), ETEC-like (2), AdhF-*Ec* (1)	05/2002–10/2019	22	EDEC (17), ETEC (5)	07/2009–09/2012
Farm 2	69	feces (43), isolate (22), intestine + feces (4)	78	ETEC (53), ETEC-like (7), EDEC (6), AEEC (6), AdhF-*Ec* (3), ETEC/STEC (2), STEC (1)	06/2004–02/2021	24	ETEC (24)	07/2013–02/2018
Farm 3	25	isolate (23), feces (2)	26	ETEC/STEC (14), ETEC (4), EDEC (3), ETEC-like (3), STEC (2)	10/2014–04/2019	16	ETEC/STEC (11), EDEC (2), ETEC (1), ETEC-like (1), STEC (1)	10/2014–04/2019

* VAGs = virulence-associated genes; InPEC = positive for at least one of the following virulence factors (and encoding genes): F4 (*faeG*), F5 (*fanA*), F6 (*fasA*), F18 (*fedA*) and F41 (*fimF41a*) (adhesive fimbriae); LT-Ia, LT-Ib (*eltB-Ip*), ST-Ia (*estap*) and ST-II (*estb*) (heat-labile/stable enterotoxins); stx2, including stx2e (*stx2*) (Shiga toxin) or intimin (*eae*) in a modified multiplex PCR [30]. ** Pathotypes: AdhF-*Ec*, positive for at least one adhesive fimbriae gene (*faeG, fanA, fasA, fedA* and *fimF41a*); AEEC, positive for *eae*; EDEC, positive for *fedA* and *stx2*; ETEC, positive for at least one adhesive fimbriae gene (*faeG, fanA, fasA, fedA* and *fimF41a*) and at least one enterotoxin gene (*eltB-Ip, estap* and *estb*); ETEC-like, positive for at least one enterotoxin gene (*eltB-Ip, estap* and *estb*); ETEC/STEC, positive for at least one adhesive fimbriae gene (*faeG, fanA, fasA, fedA* and *fimF41a*) and at least one enterotoxin gene (*eltB-Ip, estap* and *estb*) and *stx2*; STEC, positive for *stx2*.

**Table 2:** Characteristics of 87 (27/44/16) *Escherichia coli* isolates, sorted by Farms 1–3, MLST and date of isolation.

Strain ID	Source *	Date of Isolation	VAGs **	Pathotype	MLST	Clonotype	Genoserotype ***	Phylogroup	*mcr* Gene/*pmrB* Mutation	Colistin MIC (µg/mL)
**Farm 1 (*n* = 27)**										
IHIT46527	Feces	06/2005	*fedAab*, *stx2e*	EDEC	1	2-54	O139:H1	D	-	1
IHIT48337	Feces	12/2005	*fedAab*, *stx2e*	EDEC	1	2-54	Ont:H1	D	-	8
IHIT46528	Feces	01/2006	*fedAab*, *stx2e*	EDEC	1	2-54	O139:H1	D	-	4
IHIT46530	Feces	07/2009	*fedAab*, *stx2e*	EDEC	1	2-54	O139:H1	D	*mcr-1.1*	8
IHIT45339	Feces	08/2009	*fedAab*, *stx2e*	EDEC	1	2-54	O139:H1	D	*mcr-1.1*	8
IHIT46531	Feces	12/2009	*fedAab*, *stx2e*	EDEC	1	2-54	O139:H1	D	*mcr-1.1*	4
IHIT46532	Feces	01/2010	*fedAab*, *stx2e*	EDEC	1	2-54	O139:H1	D	*mcr-1.1*	8
IHIT46533	Feces	03/2010	*fedAab*, *stx2e*	EDEC	1	2-54	O139:H1	D	*mcr-1.1*	8
IHIT48339	Feces	04/2010	*fedAab*, *stx2e*	EDEC	1	2-54	O139:H1	D	*mcr-1.1*	8
IHIT32406	Feces	06/2010	*fedAab*, *stx2e*	EDEC	1	2-54	O139:H1	D	*mcr-1.1*	8
IHIT48341	Feces	06/2010	*fedAab*, *stx2e*	EDEC	1	2-54	O139:H1	D	*mcr-1.1*	8
IHIT45342	Feces	07/2011	*fedAab*, *stx2e*	EDEC	1	2-54	O139:H1	D	*mcr-1.1*	8
IHIT46538	Feces	09/2012	*fedAab*, *stx2e*	EDEC	1	2-54	O139:H1	D	*mcr-1.1*	8
IHIT46553	Isolate	10/2019	*fedAab*, *stx2e*	EDEC	1	2-54	O139:H1	D	-	0.5
IHIT52949	Feces	06/2011	*estb*, *estap*, *fedAac*	ETEC	23	4-54	O8:H17	C	-	0.25
IHIT48327	Feces	04/2004	*estb*, *eltB-Ip*, *fedAac*	ETEC	42	28-65	O147:H14	D	*pmrB* V161G	2
IHIT48328	Feces	07/2004	*estb*, *estap ^#^*, *fedAac*	ETEC	42	28-65	Ont:H14	D	*pmrB* V161G	0.5
IHIT48326	Feces	05/2002	*estb*, *eltB-Ip*, *faeGac*	ETEC	100	27-0	O149:H10	A	-	1
IHIT52948	Feces	02/2008	*estb*, *eltB-Ip*, *faeGac*	ETEC	100	27-0	O149:H10	A	-	0.25
IHIT45341	Feces	07/2011	*estb*, *eltB-Ip*, *faeGac*	ETEC	100	27-65	O149:H10	A	*mcr-1.1*	8
IHIT48351	Feces	12/2015	*estb ^#^*, *eltB-Ip*, *faeGac*	ETEC	100	27-0	Ont:H10	A	-	0.5
IHIT46534	Feces	06/2010	*estb*, *estap*, *fedAac*	ETEC	131	40-683	O25:H4	B2	*mcr-1.1*	4
IHIT48340	Feces	06/2010	*estb*, *estap*, *fedAac*	ETEC	131	40-683	O25:H4	B2	*mcr-1.1*	4
IHIT48343	Feces	06/2011	*estb*, *estap ^#^*, *fedAac*	ETEC	131	40-683	O25:H4	B2	*mcr-1.1*	8
IHIT52950	Feces	07/2012	*fedA*	AdhF-*Ec*	641	6-289	O121:H10	B1	-	0.5
IHIT48325	Feces	05/2002	*stx2e*	STEC	710	153-1582	Ont:H30	A	-	0.5
IHIT52947	Feces	12/2007	*stx2e*	STEC	12009	11-23	O142:H27	A	-	0.5
**Farm 2 (*n* = 44)**										
IHIT48329	Feces	07/2004	*fedAab*, *stx2e*	EDEC	1	2-54	O139:H1	D	-	0.5
IHIT48336	Feces	08/2005	*fedAab*, *stx2e*	EDEC	1	2-54	O139:H1	D	-	8
IHIT48354	Int + Fec	11/2020	*fedAab*	AdhF-*Ec*	1	2-54	O139:H1	D	-	0.5
IHIT48331	Feces	10/2004	*estb ^#^*	ETEC-like	10	11-54	O163:H10	A	-	0.5
IHIT46535	Feces	02/2011	*estb*, *estap*, *fedAac*	ETEC	10	11-24	O141:H4	A	-	0.5
IHIT46540	Isolate	07/2013	*estb*, *estap*, *fedAac*	ETEC	10	11-24	O141:H4	A	-	0.25
IHIT23335	Feces	07/2013	*estb*, *estap*, *fedAac*, *stx2e*	ETEC/STEC	10	11-24	O141:H4	A	-	0.25
IHIT48348	Feces	11/2014	*estb*	ETEC-like	10	11-45	Ont:H6	A	-	8
IHIT46541	Feces	11/2014	*estb*, *estap ^#^*, *fedAac*	ETEC	10	11-24	O141:H4	A	*mcr-1.1*	4
IHIT46542	Isolate	01/2015	*estb*, *estap*, *fedAac*	ETEC	10	11-24	O141:H4	A	*mcr-1.1*	4
IHIT46550	Isolate	02/2018	*estb*, *estap*, *fedAac*	ETEC	10	11-24	O141:H4	A	*mcr-1.1*	4
IHIT48346	Feces	12/2012	*eae*	AEEC	20	4-25	Ont:H49	B1	-	0.5
IHIT48330	Feces	07/2004	*eae*	AEEC	29	4-24	O123:H11	B1	-	0.5
IHIT48333	Feces	12/2004	*estb*, *eltB-Ip*, *fedAac*	ETEC	42	28-65	O147:H14	D	*pmrB* V161G	4
IHIT48334	Feces	12/2004	*fedAac*	AdhF-*Ec*	42	28-65	O147:H14	D	*pmrB* V161G	4
IHIT48342	Feces	06/2010	*eae ^#^*	AEEC	93	11-0	O5:H4	A	-	0.25
IHIT46526	Feces	06/2004	*estb*, *estap*, *eltB-Ip*, *faeGac*	ETEC	100	27-0	O149:H10	A	-	1
IHIT48332	Feces	10/2004	*estb*, *eltB-Ip*, *faeGac*	ETEC	100	27-0	O149:H10	A	-	0.5
IHIT48335	Feces	03/2005	*estb*, *estap*, *eltB-Ip*, *faeGac*	ETEC	100	27-0	O149:H10	A	-	0.25
IHIT46529	Feces	02/2006	*estb*, *estap*, *eltB-Ip*, *faeGac*	ETEC	100	27-0	O149:H10	A	-	8
IHIT48338	Feces	08/2008	*estb*, *eltB-Ip*, *faeGac*	ETEC	100	27-0	Ont:H10	A	-	4
IHIT46536	Feces	11/2011	*estb*, *estap*, *eltB-Ip*, *faeGac*	ETEC	100	27-0	O149:H10	A	-	0.5
IHIT46537	Feces	01/2012	*estb*, *estap ^#^*, *eltB-Ip*, *faeGac*	ETEC	100	27-0	O149:H10	A	-	0.5
IHIT45353	Isolate	07/2013	*estb*, *estap*, *eltB-Ip*, *faeGac*	ETEC	100	27-0	O149:H10	A	*mcr-1.1*	8
IHIT46539	Isolate	07/2013	*estb*, *estap*, *eltB-Ip*, *faeGac*	ETEC	100	27-0	O149:H10	A	-	0.25
IHIT25408	Isolate	03/2014	*estb*, *estap*, *eltB-Ip*, *faeGac*	ETEC	100	27-0	Ont:H10	A	*mcr-1.1*	0.5
IHIT48347	Isolate	05/2014	*estb*, *estap*, *eltB-Ip*, *faeGac*	ETEC	100	27-0	O149:H10	A	*mcr-1.1*	8
IHIT27622	Isolate	10/2014	*estb*, *estap*, *eltB-Ip*, *faeGac*	ETEC	100	27-0	O149:H10	A	*mcr-1.1*	8
IHIT45399	Isolate	01/2015	*estb*, *estap*, *eltB-Ip*, *faeGac*	ETEC	100	27-0	O149:H10	A	*mcr-1.1*	8
IHIT48349	Isolate	07/2015	*estb*, *estap*, *eltB-Ip*, *faeGac*	ETEC	100	27-0	Ont:H10	A	*mcr-1.1*	8
IHIT45401	Isolate	09/2015	*estb*, *estap*, *eltB-Ip*, *faeGac*	ETEC	100	27-0	O149:H10	A	*mcr-1.1*	8
IHIT46543	Isolate	09/2015	*estb*, *estap*, *eltB-Ip*, *faeGac*	ETEC	100	27-0	O149:H10	A	*mcr-1.1*	4
IHIT46546	Isolate	09/2016	*estb*, *estap*, *eltB-Ip*, *faeGac*	ETEC	100	27-0	O149:H10	A	*mcr-1.1*	8
IHIT45407	Isolate	08/2017	*estb*, *estap*, *eltB-Ip*, *faeGac*	ETEC	100	27-0	O149:H10	A	*mcr-1.1*	8
IHIT36144	Isolate	01/2018	*estb*, *estap*, *eltB-Ip*, *faeGac*	ETEC	100	27-0	O149:H10	A	*mcr-1.1*	8
IHIT36146	Isolate	01/2018	*estb*, *estap*, *eltB-Ip*, *faeGac*	ETEC	100	27-0	O149:H10	A	*mcr-1.1*	8
IHIT36426	Isolate	02/2018	*estb*, *estap*, *eltB-Ip*, *faeGac*	ETEC	100	27-0	O149:H10	A	*mcr-1.1*	8
IHIT36427	Isolate	02/2018	*estb*, *estap*, *eltB-Ip*, *faeGac*	ETEC	100	27-0	O149:H10	A	*mcr-1.1*	8
IHIT48355	Int + Fec	11/2020	*estb ^#^*	ETEC-like	641	6-832	O45:H10	B1	-	2
IHIT48358	Feces	02/2021	*estb*	ETEC-like	641	6-289	O115:H10	B1	-	0.25
IHIT32748	Isolate	09/2016	*eae*	AEEC	793	168-555	O49:H10	A	-	0.5
IHIT48344	Feces	12/2011	*eae*	AEEC	799	84-305	O108:H9	E	-	0.25
IHIT48356	Int + Fec	11/2020	*stx2e*	STEC	955	2-65	O139:H1	D	-	0.5
IHIT48350	Feces	09/2015	*estb*	ETEC-like	2944	224-1082	O17/O77:H28	D	-	1
**Farm 3 (*n* = 16)**										
IHIT34769	Isolate	06/2017	*fedAab*, *stx2e*	EDEC	1	2-54	O139:H1	D	*mcr-1.1*	4
IHIT48353	Isolate	06/2017	*faeGac*, *stx2e*	EDEC	1	2-54	Ont:H1	D	*mcr-1.1*	8
IHIT47062	Isolate	10/2017	*fedAab*, *stx2e*	EDEC	1	2-54	O139:H1	D	-	1
IHIT48352	Feces	12/2016	*stx2e*	STEC	10	11-23	Ont.:H32	A	*mcr-1.1*	8
IHIT39537	Isolate	04/2019	*estb*	ETEC-like	10	11-2594	O35:H6	A	*mcr-4.8*	4
IHIT47044	Isolate	10/2014	*estb*, *estap*, *fedAac*, *stx2e*	ETEC/STEC	86	6-32	Ont:H10	B1	*mcr-1.1*	0.5
IHIT47045	Isolate	10/2014	*estb*, *estap*, *fedAac*, *stx2 ^#^*	ETEC/STEC	86	6-32	O86:H10	B1	-	1
IHIT47046	Isolate	06/2015	*estb*, *estap*, *fedAac*, *stx2e*	ETEC/STEC	86	6-32	O86:H10	B1	*mcr-1.1*	4
IHIT47048	Isolate	06/2015	*estb*, *estap*, *fedAac*, *stx2e*	ETEC/STEC	86	6-32	O86:H10	B1	-	0.25
IHIT47056	Isolate	06/2016	*estb*, *estap*, *fedAac*, *stx2e*	ETEC/STEC	86	6-32	Ont:H10	B1	*mcr-1.1*	4
IHIT47057	Isolate	11/2016	*estb*, *estap*, *fedAac*	ETEC	86	6-32	O86:H10	B1	-	0.5
IHIT34315	Isolate	04/2017	*estb*, *estap*, *fedAac*, *stx2e*	ETEC/STEC	86	6-32	Ont:H10	B1	*mcr-1.1*	4
IHIT47060	Isolate	02/2017	*estb*, *eltB-Ip*, *faeGac*	ETEC	90	4-54	O8:H19	C	-	0.5
IHIT47047	Isolate	06/2015	*estb*, *eltB-Ip*, *faeGac*	ETEC	100	27-0	O149:H10	A	*mcr-1.1*	8
IHIT47065	Isolate	07/2018	*estb*	ETEC-like	118	4-331	O15:H45	E	-	0.5
IHIT47072	Isolate	03/2019	*estb*	ETEC-like	162	65-32	O8:H19	B1	-	0.25

* Int + Fec = intestine and feces. ** VAGs = virulence-associated genes for InPEC: *faeGac*, *fedAab* and *fedAac* (adhesive fimbriae); *eltB-Ip*, *estap* and *estb* (heat-labile/stable enterotoxins); *stx2* (Shiga toxin) or *eae* (intimin). VAGs marked with ^#^ were positive in the PCR but negative for the respective virulence gene according to whole genome data. *** Ont = O not typeable.

## Data Availability

All relevant data are provided in the paper and its Supplements. Raw sequence reads of 87 *E. coli* genomes are provided under NCBI Bioproject ID PRJNA916215. Further raw data can be made available on reasonable request.

## Supplementary Materials

The following supporting information can be downloaded at: https://www.mdpi.com/article/10.3390/microorganisms12040729/s1, Table S1: 87 *E. coli* were analyzed to determine the location of virulence genes associated with intestinal pathogenic *E. coli*. Isolates are sorted by farms, sequence type (ST), and date of isolation; Table S2: Distribution of virulence-associated genes among 87 *E. coli* isolates from swine, sorted by farms, sequence types (ST), and date of isolation. Only genes that were at least present in one of the isolates with an identity of >90% (VirulenceFinder 2.0 and BacWGSTdb), are listed; Table S3: Pathotypes, sequence types (STs), resistance genes and chromosomal point mutations related to antimicrobial resistance of 87 representative whole genome-sequenced *E. coli* isolates, sorted by farms, STs, and date of isolation.
