# Supplementary material for: Repeated Occurrence of Mobile Colistin Resistance Gene-Carrying Plasmids in Pathogenic Escherichia coli from German Pig Farms
Source: Microorganisms. 2024 Apr 3;12(4):729. doi: 10.3390/microorganisms12040729 (PMC11052496; doi:10.3390/microorganisms12040729)
Supplement: Supplementary file 1 [file microorganisms-12-00729-s001.zip › Table S1.pdf]

**Table S1**: 87 *E. coli* were analyzed to determine the location of virulence genes associated with intestinal pathogenic *E. coli*. Isolates are sorted by farms, sequence type (ST), and date of isolation.

| Strain ID | Date of isolation | ST | Pathotype* | VAG** (adhesin) | Reference plasmid, adhesin (NCBI Reference Sequence) | VAGs** (toxin) | Reference plasmid, for one or several toxin (NCBI Reference Sequence) |
|---|---|---|---|---|---|---|---|
| **Farm 1 (*n* = 27)** | | | | | | | |
| IHIT46527 | 06/2005 | 1 | EDEC | *fedAab* | p15ODTXV (MG904998.1) | *stx2e* | - |
| IHIT48337 | 12/2005 | 1 | EDEC | *fedAab* | p1713-1 (CP031766.1) | *stx2e* | - |
| IHIT46528 | 01/2006 | 1 | EDEC | *fedAab* | p15ODTXV (MG904998.1) | *stx2e* | - |
| IHIT46530 | 07/2009 | 1 | EDEC | *fedAab* | p15ODTXV (MG904998.1) | *stx2e* | - |
| IHIT45339 | 08/2009 | 1 | EDEC | *fedAab* | p15ODTXV (MG904998.1) | *stx2e* | - |
| IHIT46531 | 12/2009 | 1 | EDEC | *fedAab* | p15ODTXV (MG904998.1) | *stx2e* | - |
| IHIT46532 | 01/2010 | 1 | EDEC | *fedAab* | p15ODTXV (MG904998.1) | *stx2e* | - |
| IHIT46533 | 03/2010 | 1 | EDEC | *fedAab* | p15ODTXV (MG904998.1) | *stx2e* | - |
| IHIT48339 | 04/2010 | 1 | EDEC | *fedAab* | p15ODTXV (MG904998.1) | *stx2e* | - |
| IHIT32406 | 06/2010 | 1 | EDEC | *fedAab* | p15ODTXV (MG904998.1) | *stx2e* | - |
| IHIT48341 | 06/2010 | 1 | EDEC | *fedAab* | p15ODTXV (MG904998.1) | *stx2e* | - |
| IHIT45342 | 07/2011 | 1 | EDEC | *fedAab* | p15ODTXV (MG904998.1) | *stx2e* | - |
| IHIT46538 | 09/2012 | 1 | EDEC | *fedAab* | p15ODTXV (MG904998.1) | *stx2e* | - |
| IHIT46553 | 10/2019 | 1 | EDEC | *fedAab* | p15ODTXV (MG904998.1) | *stx2e* | - |
| IHIT52949 | 06/2011 | 23 | ETEC | *fedAac* | p15ODTXV (MG904998.1) | *estb, estap* | p15ODTXV (MG904998.1) |
| IHIT48327 | 04/2004 | 42 | ETEC | *fedAac* | p15ODTXV (MG904998.1) | *estb, eltB-Ip* | p14ODTX (MG904993.1) |
| IHIT48328 | 07/2004 | 42 | ETEC | *fedAac* | pCV839-06-p2 (CP025752.1) | *estb, estap*[#] | pCV839-06-p2 (CP025752.1) |
| IHIT48326 | 05/2002 | 100 | ETEC | *faeGac* | pUMNK88_K88 (CP002730.1) | *estb, eltB-Ip* | p14ODTX (MG904993.1) |
| IHIT52948 | 02/2008 | 100 | ETEC | *faeGac* | pUMNK88_K88 (CP002730.1) | *estb, eltB-Ip* | pUMNK88_Ent (NC_017640.1) |
| IHIT45341 | 07/2011 | 100 | ETEC | *faeGac* | p1713-1 (CP031766.1) | *estb, eltB-Ip* | pUMNK88_Ent (NC_017640.1) |
| IHIT48351 | 12/2015 | 100 | ETEC | *faeGac* | p14ODK88 (MG904991.1) | *estb*[#]*, eltB-Ip* | pUMNK88_Ent (NC_017640.1) |
| IHIT46534 | 06/2010 | 131 | ETEC | *fedAac* | p15ODTXV (MG904998.1) | *estb, estap* | p15ODTXV (MG904998.1) |
| IHIT48340 | 06/2010 | 131 | ETEC | *fedAac* | p15ODTXV (MG904998.1) | *estb, estap* | p15ODTXV (MG904998.1) |
| IHIT48343 | 06/2011 | 131 | ETEC | *fedAac* | p15ODTXV (MG904998.1) | *estb, estap*[#] | p15ODTXV (MG904998.1) |
| IHIT52950 | 07/2012 | 641 | AdhF-*Ec* | *fedA* | pCV839-06-p2 (CP025752.1) | - | - |

| Strain ID | Date of isolation | ST | Pathotype* | VAG** (adhesin) | Reference plasmid, adhesin (NCBI Reference Sequence) | VAGs** (toxin) | Reference plasmid, for one or several toxin (NCBI Reference Sequence) |
|---|---|---|---|---|---|---|---|
| IHIT48325 | 05/2002 | 710 | STEC | - | - | *stx2e* | - |
| IHIT52947 | 12/2007 | 12009 | STEC | - | - | *stx2e* | - |
| **Farm 2 (*n* = 44)** | | | | | | | |
| IHIT48329 | 07/2004 | 1 | EDEC | *fedAab* | p15ODTXV (MG904998.1) | *stx2e* | - |
| IHIT48336 | 08/2005 | 1 | EDEC | *fedAab* | p15ODTXV (MG904998.1) | *stx2e* | - |
| IHIT48354 | 11/2020 | 1 | AdhF-*Ec* | *fedAab* | p1713-1 (CP031766.1) | - | - |
| IHIT48331 | 10/2004 | 10 | ETEC-like | - | - | *estb*# | - |
| IHIT46535 | 02/2011 | 10 | ETEC | *fedAac* | p15ODTXV (MG904998.1) | *estb, estap* | p15ODTXV (MG904998.1) |
| IHIT46540 | 07/2013 | 10 | ETEC | *fedAac* | p15ODTXV (MG904998.1) | *estb, estap* | p15ODTXV (MG904998.1) |
| IHIT23335 | 07/2013 | 10 | ETEC/STEC | *fedAac* | p15ODTXV (MG904998.1) | *stx2e, estb, estap* | p2454 (MG948333.1) |
| IHIT48348 | 11/2014 | 10 | ETEC-like | - | - | *estb* | - |
| IHIT46541 | 11/2014 | 10 | ETEC | *fedAac* | p15ODTXV (MG904998.1) | *estb, estap*# | p15ODTXV (MG904998.1) |
| IHIT46542 | 01/2015 | 10 | ETEC | *fedAac* | p15ODTXV (MG904998.1) | *estb, estap* | p15ODTXV (MG904998.1) |
| IHIT46550 | 02/2018 | 10 | ETEC | *fedAac* | p15ODTXV (MG904998.1) | *estb, estap* | p15ODTXV (MG904998.1) |
| IHIT48346 | 12/2012 | 20 | AEEC | *eae* | - | - | - |
| IHIT48330 | 07/2004 | 29 | AEEC | *eae* | - | - | - |
| IHIT48333 | 12/2004 | 42 | ETEC | *fedAac* | p35K (CP022728.1) | *estb, eltB-Ip* | p35K (CP022728.1) |
| IHIT48334 | 12/2004 | 42 | AdhF-*Ec* | *fedAac* | p14ODV (MG904994.1) | - | - |
| IHIT48342 | 06/2010 | 93 | AEEC | *eae*# | - | - | - |
| IHIT46526 | 06/2004 | 100 | ETEC | *faeGac* | p14ODK88 (MG904991.1) | *estb, estap, eltB-Ip* | pUMNK88_Ent (NC_017640.1) |
| IHIT48332 | 10/2004 | 100 | ETEC | *faeGac* | p15ODTXV (MG904998.1) | *estb, eltB-Ip* | pGMI14-004_1 (CP028195.1) |
| IHIT48335 | 03/2005 | 100 | ETEC | *faeGac* | pUMNK88_K88 (CP002730.1) | *estb, estap, eltB-Ip* | pGMI14-004_1 (CP028195.1) |
| IHIT46529 | 02/2006 | 100 | ETEC | *faeGac* | pUMNK88_K88 (CP002730.1) | *estb, estap, eltB-Ip* | pUMNK88_Ent (NC_017640.1) |
| IHIT48338 | 08/2008 | 100 | ETEC | *faeGac* | pUMNK88_K88 (CP002730.1) | *estb, eltB-Ip* | pUMNK88_Ent (NC_017640.1) |
| IHIT46536 | 11/2011 | 100 | ETEC | *faeGac* | pUMNK88_K88 (CP002730.1) | *estb, estap, eltB-Ip* | pUMNK88_Ent (NC_017640.1) |
| IHIT46537 | 01/2012 | 100 | ETEC | *faeGac* | pUMNK88_K88 (CP002730.1) | *estb, estap*#*, eltB-Ip* | pUMNK88_Ent (NC_017640.1) |
| IHIT45353 | 07/2013 | 100 | ETEC | *faeGac* | pUMNK88_K88 (CP002730.1) | *estb, estap, eltB-Ip* | pUMNK88_Ent (NC_017640.1) |
| IHIT46539 | 07/2013 | 100 | ETEC | *faeGac* | pUMNK88_K88 (CP002730.1) | *estb, estap, eltB-Ip* | pUMNK88_Ent (NC_017640.1) |
| IHIT25408 | 03/2014 | 100 | ETEC | *faeGac* | p14ODK88 (MG904991.1) | *estb, estap, eltB-Ip* | pUMNK88_Ent (NC_017640.1) |

| Strain ID | Date of isolation | ST | Pathotype* | VAG** (adhesin) | Reference plasmid, adhesin (NCBI Reference Sequence) | VAGs** (toxin) | Reference plasmid, for one or several toxin (NCBI Reference Sequence) |
|---|---|---|---|---|---|---|---|
| IHIT48347 | 05/2014 | 100 | ETEC | *faeGac* | p14ODK88 (MG904991.1) | *estb, estap, eltB-Ip* | pUMNK88_Ent (NC_017640.1) |
| IHIT27622 | 10/2014 | 100 | ETEC | *faeGac* | p14ODK88 (MG904991.1) | *estb, estap, eltB-Ip* | pGMI14-004_1 (CP028195.1) |
| IHIT45399 | 01/2015 | 100 | ETEC | *faeGac* | pUMNK88_K88 (CP002730.1) | *estb, estap, eltB-Ip* | pUMNK88_Ent (NC_017640.1) |
| IHIT48349 | 07/2015 | 100 | ETEC | *faeGac* | p14ODK88 (MG904991.1) | *estb, estap, eltB-Ip* | pUMNK88_Ent (NC_017640.1) |
| IHIT45401 | 09/2015 | 100 | ETEC | *faeGac* | p14ODK88 (MG904991.1) | *estb, estap, eltB-Ip* | pUMNK88_Ent (NC_017640.1) |
| IHIT46543 | 09/2015 | 100 | ETEC | - | p15ODTXV (MG904998.1) | - | |
| IHIT46546 | 09/2016 | 100 | ETEC | *faeGac* | pUMNK88_K88 (CP002730.1) | *estb, estap, eltB-Ip* | pGMI14-004_1 (CP028195.1) |
| IHIT45407 | 08/2017 | 100 | ETEC | *faeGac* | pUMNK88_K88 (CP002730.1) | *estb, estap, eltB-Ip* | pUMNK88_Ent (NC_017640.1) |
| IHIT36144 | 01/2018 | 100 | ETEC | *faeGac* | pUMNK88_K88 (CP002730.1) | *estb, estap, eltB-Ip* | pGMI14-004_1 (CP028195.1) |
| IHIT36146 | 01/2018 | 100 | ETEC | *faeGac* | pUMNK88_K88 (CP002730.1) | *estb, estap, eltB-Ip* | pGMI14-004_1 (CP028195.1) |
| IHIT36426 | 02/2018 | 100 | ETEC | *faeGac* | pUMNK88_K88 (CP002730.1) | *estb, estap, eltB-Ip* | pGMI14-004_1 (CP028195.1) |
| IHIT36427 | 02/2018 | 100 | ETEC | *faeGac* | pUMNK88_K88 (CP002730.1) | *estb, estap, eltB-Ip* | pGMI14-004_1 (CP028195.1) |
| IHIT48355 | 11/2020 | 641 | ETEC-like | - | - | *estb*[#] | - |
| IHIT48358 | 02/2021 | 641 | ETEC-like | - | - | *estb* | - |
| IHIT32748 | 09/2016 | 793 | AEEC | *eae* | - | - | - |
| IHIT48344 | 12/2011 | 799 | AEEC | *eae* | - | - | - |
| IHIT48356 | 11/2020 | 955 | STEC | - | - | *stx2e* | - |
| IHIT48350 | 09/2015 | 2944 | ETEC-like | - | - | *estb* | pOX38 (MF370216.1) |
| **Farm 3 (*n* = 16)** | | | | | | | |
| IHIT34769 | 06/2017 | 1 | EDEC | *fedAab* | p15ODTXV (MG904998.1) | *stx2e* | - |
| IHIT48353 | 06/2017 | 1 | EDEC | *faeGac* | - | *stx2e* | - |
| IHIT47062 | 10/2017 | 1 | EDEC | *fedAab* | p15ODTXV (MG904998.1) | *stx2e* | - |
| IHIT48352 | 12/2016 | 10 | STEC | - | - | *stx2e* | - |
| IHIT39537 | 04/2019 | 10 | ETEC-like | - | - | *estb* | - |
| IHIT47044 | 10/2014 | 86 | ETEC/STEC | *fedAac* | p15ODTXV (MG904998.1) | *stx2e, estb, estap* | p15ODTXV (MG904998.1) |
| IHIT47045 | 10/2014 | 86 | ETEC/STEC | *fedAac* | p15ODTXV (MG904998.1) | *stx2e, estb, estap* | p15ODTXV (MG904998.1) |
| IHIT47046 | 06/2015 | 86 | ETEC/STEC | *fedAac* | p15ODTXV (MG904998.1) | *stx2e, estb, estap* | p15ODTXV (MG904998.1) |
| IHIT47048 | 06/2015 | 86 | ETEC/STEC | *fedAac* | p15ODTXV (MG904998.1) | *stx2e, estb, estap* | p15ODTXV (MG904998.1) |
| IHIT47056 | 06/2016 | 86 | ETEC/STEC | *fedAac* | p15ODTXV (MG904998.1) | *stx2e, estb, estap* | p15ODTXV (MG904998.1) |

| Strain ID | Date of isolation | ST | Pathotype* | VAG** (adhesin) | Reference plasmid, adhesin (NCBI Reference Sequence) | VAGs** (toxin) | Reference plasmid, for one or several toxin (NCBI Reference Sequence) |
|---|---|---|---|---|---|---|---|
| IHIT47057 | 11/2016 | 86 | ETEC | *fedAac* | p15ODTXV (MG904998.1) | *estb, estap* | p15ODTXV (MG904998.1) |
| IHIT34315 | 04/2017 | 86 | ETEC/STEC | *fedAac* | p15ODTXV (MG904998.1) | *stx2e, estb, estap* | p15ODTXV (MG904998.1) |
| IHIT47060 | 02/2017 | 90 | ETEC | *faeGac* | pUMNK88_K88 (CP002730.1) | *estb, eltB-Ip* | p14ODTX (MG904993.1) |
| IHIT47047 | 06/2015 | 100 | ETEC | *faeGac* | pUMNK88_K88 (CP002730.1) | *estb, eltB-Ip* | p14ODTX (MG904993.1) |
| IHIT47065 | 07/2018 | 118 | ETEC-like | - | - | *estb* | pOX38 (MF370216.1) |
| IHIT47072 | 03/2019 | 162 | ETEC-like | - | - | *estb* | - |

*Virulence-associated genes (VAGs) marked with # were positive in the PCR but negative for the respective virulence gene according to whole genome data. **Pathotypes: AdhF-*Ec*, positive for at least one adhesive fimbriae gene (*faeG, fanA, fasA, fedA, fimF41a*); AEEC, positive for *eae*; EDEC, positive for *fedA* and *stx2*; ETEC, positive for at least one adhesive fimbriae gene (*faeG, fanA, fasA, fedA, fimF41a*) and at least one enterotoxin gene (*eltB-Ip, estap, estb*); ETEC-like, positive for at least one enterotoxin gene (*eltB-Ip, estap, estb*); ETEC/STEC, positive for at least one adhesive fimbriae gene (*faeG, fanA, fasA, fedA, fimF41a*) and at least one enterotoxin gene (*eltB-Ip, estap, estb*) and *stx2*; STEC, positive for *stx2*.
