# Supplementary material for: Repeated Occurrence of Mobile Colistin Resistance Gene-Carrying Plasmids in Pathogenic Escherichia coli from German Pig Farms
Source: Microorganisms. 2024 Apr 3;12(4):729. doi: 10.3390/microorganisms12040729 (PMC11052496; doi:10.3390/microorganisms12040729)
Supplement: Supplementary file 1 [file microorganisms-12-00729-s001.zip › Table S3.pdf]

Table S3: Pathotypes, sequence types (STs), resistance genes and chromosomal point mutations related to antimicrobial resistance of 87 representative whole genome sequenced *E. coli* isolates, sorted by farms, STs, and date of isolation.

Strain ID	Date of isolation	Pathotype*	ST	*mcr* gene	Further resistance genes	Chromosomal point mutation
**Farm 1 (*n* = 27)**	05/2002 - 10/2019	EDEC (51.9%), ETEC (37.0%), STEC (7.4%), AdhF-*Ec* (3.7%)	ST1 (51.9%), ST100 (14.8%), ST131 (11.1%), ST42 (7.4%), ST23 (3.7%), ST641 (3.7%), ST710 (3.7%), ST12009 (3.7%)	*mcr-1.1* (51.9%)	*sul2* (85.2%), *aph(3``)-Ib* (77.8%), *aph(6)-Id* (77.8%), *bla*$_{TEM-1B}$ (77.8%), *tet(A)* (74.1%), *catA1* (33.3%), *aadA2b* (29.6%), *sul3* (29.6%), *tet(C)* (29.6%), *aac(3)-IV* (25.9%), *aph(4)-Ia* (25.9%), *dfrA1* (18.5%), *aadA1* (14.8%), *dfrA14* (14.8%), *sul1* (14.8%), *tet(B)* (11.1%), *aadA24* (7.4%), *aadA13* (3.7%), *bla*$_{TEM-1A}$ (3.7%), *cmlA1* (3.7%)	*parE* I529L (11.1%), *pmrB* V161G (7.4%), *gyrA* S83L (3.7%)
IHIT46527	06/2005	EDEC	1	-	-	-
IHIT48337	12/2005	EDEC	1	-	*aph(3``)-Ib, aph(6)-Id, bla*$_{TEM-1B}$, *sul2, tet(A)*	-
IHIT46528	01/2006	EDEC	1	-	*aph(3``)-Ib, aph(6)-Id, bla*$_{TEM-1B}$, *sul2, tet(A)*	-
IHIT46530	07/2009	EDEC	1	*mcr-1.1*	*aph(3``)-Ib, aph(6)-Id, bla*$_{TEM-1B}$, *sul2, tet(A), tet(C)*	-
IHIT45339	08/2009	EDEC	1	*mcr-1.1*	*aph(3``)-Ib, aph(6)-Id, bla*$_{TEM-1B}$, *sul2, tet(A), tet(C)*	-
IHIT46531	12/2009	EDEC	1	*mcr-1.1*	*aac(3)-IV, aadA2b, aph(3``)-Ib, aph(4)-Ia, aph(6)-Id, bla*$_{TEM-1B}$, *catA1, sul2, sul3, tet(A), tet(C)*	-
IHIT46532	01/2010	EDEC	1	*mcr-1.1*	*aph(3``)-Ib, aph(6)-Id, bla*$_{TEM-1B}$, *sul2, tet(A), tet(C)*	-
IHIT46533	03/2010	EDEC	1	*mcr-1.1*	*aac(3)-IV, aadA2b, aph(3``)-Ib, aph(4)-Ia, aph(6)-Id, bla*$_{TEM-1B}$, *catA1, sul2, sul3, tet(A), tet(C)*	-
IHIT48339	04/2010	EDEC	1	*mcr-1.1*	*aac(3)-IV, aadA2b, aph(3``)-Ib, aph(4)-Ia, aph(6)-Id, bla*$_{TEM-1B}$, *catA1, sul2, sul3, tet(A), tet(C)*	-
IHIT32406	06/2010	EDEC	1	*mcr-1.1*	*aac(3)-IV, aadA2b, aph(3``)-Ib, aph(4)-Ia, aph(6)-Id, bla*$_{TEM-1B}$, *catA1, sul2, sul3, tet(A), tet(C)*	-
IHIT48341	06/2010	EDEC	1	*mcr-1.1*	*aac(3)-IV, aadA2b, aph(3``)-Ib, aph(4)-Ia, aph(6)-Id, bla*$_{TEM-1B}$, *catA1, sul2, sul3, tet(A), tet(C)*	-
IHIT45342	07/2011	EDEC	1	*mcr-1.1*	*aac(3)-IV, aadA2b, aph(3``)-Ib, aph(4)-Ia, aph(6)-Id, bla*$_{TEM-1B}$, *catA1, sul2, sul3, tet(A)*	-
IHIT46538	09/2012	EDEC	1	*mcr-1.1*	*aac(3)-IV, aadA2b, aph(3``)-Ib, aph(4)-Ia, aph(6)-Id, bla*$_{TEM-1B}$, *catA1, sul2, sul3, tet(A)*	-
IHIT46553	10/2019	EDEC	1	-	*bla*$_{TEM-1B}$, *dfrA1, sul2*	-
IHIT52949	06/2011	ETEC	23	-	*aadA1, bla*$_{TEM-1A}$, *dfrA1, sul2, tet(B)*	-
IHIT48327	04/2004	ETEC	42	-	*aph(3``)-Ib, aph(6)-Id, sul2*	*pmrB* V161G
IHIT48328	07/2004	ETEC	42	-	*aadA1*	*pmrB* V161G, *gyrA* S83L
IHIT48326	05/2002	ETEC	100	-	*aadA1, catA1, sul1*	-
IHIT52948	02/2008	ETEC	100	-	*aph(3``)-Ib, aph(6)-Id, bla*$_{TEM-1B}$, *dfrA14, sul2, tet(A)*	-
IHIT45341	07/2011	ETEC	100	*mcr-1.1*	*aadA1, aadA2b, aph(3``)-Ib, aph(6)-Id, bla*$_{TEM-1B}$, *cmlA1, catA1, sul2, sul3, dfrA1, dfrA14, tet(A)*	-

Strain ID	Date of isolation	Pathotype*	ST	*mcr* gene	Further resistance genes	Chromosomal point mutation
IHIT48351	12/2015	ETEC	100	-	*aph(3``)-Ib, aph(6)-Id, bla*$_{TEM-1B}$, *dfrA14, sul2, tet(A)*	-
IHIT46534	06/2010	ETEC	131	*mcr-1.1*	*aadA24, aph(3``)-Ib, aph(6)-Id, bla*$_{TEM-1B}$, *dfrA1, sul1, sul2, tet(A)*	*parE* I529L
IHIT48340	06/2010	ETEC	131	*mcr-1.1*	*aadA24, aph(3``)-Ib, aph(6)-Id, bla*$_{TEM-1B}$, *dfrA1, sul1, sul2, tet(A)*	*parE* I529L
IHIT48343	06/2011	ETEC	131	*mcr-1.1*	*aph(3``)-Ib, aph(6)-Id, bla*$_{TEM-1B}$, *sul2, tet(A)*	*parE* I529L
IHIT52950	07/2012	AdhF-*Ec*	641	-	*tet(B)*	-
IHIT48325	05/2002	STEC	710	-	*aadA13, aph(3``)-Ib, aph(6)-Id, bla*$_{TEM-1B}$, *sul1, sul2, tet(A)*	-
IHIT52947	12/2007	STEC	12009	-	*aph(3``)-Ib, aph(6)-Id, bla*$_{TEM-1B}$, *dfrA14, sul2, tet(A), tet(B)*	-
**Farm 2** **(*n* = 44)**	06/2004-02/2021	ETEC (63.6%), AEEC (11.4%), ETEC-like (11.4%), AdhF-*Ec* (4.6%), EDEC (4.6%), ETEC/STEC (2.3%), STEC (2.3%)	ST100 (50%), ST10 (18.2%), ST1 (6.8%), ST42 (4.6%), ST641 (4.6%), ST20 (2.3%), ST29 (2.3%), ST93 (2.3%), ST793 (2.3%), ST799 (2.3%), ST955 (2.3%), ST2944 (2.3%)	*mcr-1.1* (38.6%)	*tet(A)* (79.6%), *bla*$_{TEM-1B}$ (72.7%), *sul2* (68.2%), *aadA1* (45.5%), *aph(3``)-Ib* (43.2%), *aph(6)-Id* (43.2%), *sul1* (29.6%), *dfrA1* (20.5%), *aadA5* (18.2%), *dfrA14* (15.9%), *catB3* (13.6%), *tet(B)* (13.6%), *bla*$_{TEM-135}$ (11.4%), *catA1* (11.4%), *mph(A)* (9.1%), *aac(3)-IV* (4.6%), *aph(4)-Ia* (4.6%), *bla*$_{TEM-1A}$ (4.6%), *dfrA17* (4.6%), *sul3* (4.6%), *aadA2* (2.3%), *aadA24* (2.3%), *bla*$_{CTX-M-1}$ (2.3%), *cmlA* (2.3%), *dfrA8* (2.3%), *dfrA12* (2.3%), *qnrS1* (2.3%)	*pmrB* V161G (4.6%), *gyrA* S83L (4.6%), *parC* A56T (2.3%), *parE* I355T (2.3%)
IHIT48329	07/2004	EDEC	1	-	*aadA1, aph(3``)-Ib, aph(6)-Id, bla*$_{TEM-1B}$, *catA1, sul1, sul2, tet(A)*	-
IHIT48336	08/2005	EDEC	1	-	*aadA1, bla*$_{TEM-1A}$, *dfrA1, sul2, tet(B)*	-
IHIT48354	11/2020	AdhF-*Ec*	1	-	*aadA1, bla*$_{TEM-1B}$, *sul1, tet(A), tet(B)*	-
IHIT48331	10/2004	ETEC-like	10	-	*aph(3``)-Ib, aph(6)-Id, bla*$_{TEM-1B}$, *dfrA14, sul2, tet(A)*	-
IHIT46535	02/2011	ETEC	10	-	*aadA1, aph(3``)-Ib, aph(6)-Id, bla*$_{CTX-M-1}$, *bla*$_{TEM-135}$, *sul2, tet(A)*	-
IHIT46540	07/2013	ETEC	10	-	*aadA1, aph(3``)-Ib, aph(6)-Id, bla*$_{TEM-135}$, *sul2, tet(A)*	-
IHIT23335	07/2013	ETEC/STEC	10	-	*aadA1, aadA24, aph(3')-Ia, bla*$_{TEM-1B}$, *sul3, tet(A)*	-
IHIT48348	11/2014	ETEC-like	10	-	*aadA1, aadA2, aph(3``)-Ib, aph(6)-Id, bla*$_{TEM-1B}$, *cmlA, dfrA12, mph(A), sul3, tet(B)*	-
IHIT46541	11/2014	ETEC	10	*mcr-1.1*	*aadA1, aph(3``)-Ib, aph(6)-Id, bla*$_{TEM-135}$, *sul2, tet(A)*	-
IHIT46542	01/2015	ETEC	10	*mcr-1.1*	*aadA1, aph(3``)-Ib, aph(6)-Id, bla*$_{TEM-135}$, *sul2, tet(A)*	-
IHIT46550	02/2018	ETEC	10	*mcr-1.1*	*aadA1, aph(3``)-Ib, aph(6)-Id, bla*$_{TEM-135}$, *sul2, tet(A)*	-
IHIT48346	12/2012	AEEC	20	-	*aadA1, dfrA1, sul2, tet(B)*	-
IHIT48330	07/2004	AEEC	29	-	*aadA1, aadA5, bla*$_{TEM-1B}$, *mph(A), sul1, dfrA17, tet(A)*	-
IHIT48333	12/2004	ETEC	42	-	*aadA1*	*pmrB* V161G, *gyrA* S83L

Strain ID	Date of isolation	Pathotype*	ST	*mcr* gene	Further resistance genes	Chromosomal point mutation
IHIT48334	12/2004	AdhF-*Ec*	42	-	*aadA1*	*pmrB* V161G, *gyrA* S83L
IHIT48342	06/2010	AEEC	93	-	*aph(3``)-Ib, aph(6)-Id, bla*$_{TEM-1B}$*, dfrA8, sul2, tet(A)*	-
IHIT46526	06/2004	ETEC	100	-	*aadA1, bla*$_{TEM-1B}$*, catA1, sul1, tet(A)*	-
IHIT48332	10/2004	ETEC	100	-	*aac(3)-IV, aph(3``)-Ib, aph(4)-Ia, aph(6)-Id, bla*$_{TEM-1B}$*, sul2, tet(A)*	-
IHIT48335	03/2005	ETEC	100	-	*aadA1, bla*$_{TEM-1B}$*, catA1, sul1, tet(A),*	-
IHIT46529	02/2006	ETEC	100	-	*aadA1, aadA5, aac(3)-IId, aph(3``)-Ib, aph(6)-Id, bla*$_{TEM-1B}$*, dfrA17, catA1, mph(A), sul1, tet(A)*	-
IHIT48338	08/2008	ETEC	100	-	*aadA1, aac(3)-IV, aph(3``)-Ib, aph(4)-Ia, aph(6)-Id, bla*$_{TEM-1B}$*, catA1, mph(A), tet(A)*	-
IHIT46536	11/2011	ETEC	100	-	*aadA5, aph(3``)-Ib, aph(6)-Id, bla*$_{TEM-1B}$*, catB3, dfrA1, dfrA14, sul1, sul2, tet(A)*	-
IHIT46537	01/2012	ETEC	100	-	*aadA5, aph(3``)-Ib, aph(6)-Id, bla*$_{TEM-1B}$*, catB3, dfrA1, dfrA14, sul1, sul2, tet(A)*	-
IHIT45353	07/2013	ETEC	100	*mcr-1.1*	*aadA5, aph(3``)-Ib, aph(6)-Id, bla*$_{TEM-1B}$*, catB3, dfrA1, dfrA14, sul1, sul2, tet(A)*	-
IHIT46539	07/2013	ETEC	100	-	*aadA5, aph(3``)-Ib, aph(6)-Id, bla*$_{TEM-1B}$*, catB3, dfrA1, dfrA14, sul1, sul2, tet(A)*	-
IHIT25408	03/2014	ETEC	100	*mcr-1.1*	*aadA5, aph(3``)-Ib, aph(6)-Id, bla*$_{TEM-1B}$*, catB3, dfrA1, dfrA14, sul1, sul2, tet(A)*	-
IHIT48347	05/2014	ETEC	100	*mcr-1.1*	*aadA5, aph(3``)-Ib, aph(6)-Id, bla*$_{TEM-1B}$*, catB3, dfrA1, dfrA14, sul1, sul2, tet(A)*	-
IHIT27622	10/2014	ETEC	100	*mcr-1.1*	*bla*$_{TEM-1B}$*, sul2, tet(A)*	-
IHIT45399	01/2015	ETEC	100	*mcr-1.1*	*bla*$_{TEM-1B}$*, sul2, tet(A)*	-
IHIT48349	07/2015	ETEC	100	*mcr-1.1*	*bla*$_{TEM-1B}$*, sul2, tet(A)*	-
IHIT45401	09/2015	ETEC	100	*mcr-1.1*	*bla*$_{TEM-1B}$*, sul2, tet(A)*	-
IHIT46543	09/2015	ETEC	100	*mcr-1.1*	*bla*$_{TEM-1B}$*, sul2, tet(A)*	-
IHIT46546	09/2016	ETEC	100	*mcr-1.1*	*bla*$_{TEM-1B}$*, sul2, tet(A)*	-
IHIT45407	08/2017	ETEC	100	*mcr-1.1*	*bla*$_{TEM-1B}$*, sul2, tet(A)*	-
IHIT36144	01/2018	ETEC	100	*mcr-1.1*	*bla*$_{TEM-1B}$*, sul2, tet(A)*	-
IHIT36146	01/2018	ETEC	100	*mcr-1.1*	*bla*$_{TEM-1B}$*, sul2, tet(A)*	-
IHIT36426	02/2018	ETEC	100	*mcr-1.1*	*bla*$_{TEM-1B}$*, sul2, tet(A)*	-
IHIT36427	02/2018	ETEC	100	*mcr-1.1*	*bla*$_{TEM-1B}$*, sul2, tet(A)*	-
IHIT48355	11/2020	ETEC-like	641	-	*tet(B)*	-
IHIT48358	02/2021	ETEC-like	641	-	*bla*$_{TEM-1B}$	-
IHIT32748	09/2016	AEEC	793	-	*aadA1, bla*$_{TEM-1B}$*, qnrS1, sul1, tet(A)*	*parC* A56T
IHIT48344	12/2011	AEEC	799	-	*aadA1, bla*$_{TEM-1A}$*, dfrA1, sul2, tet(A)*	-
IHIT48356	11/2020	STEC	955	-	*aph(3``)-Ib, aph(6)-Id, bla*$_{TEM-1B}$*, sul2, tet(B)*	-

Strain ID	Date of isolation	Pathotype*	ST	*mcr* gene	Further resistance genes	Chromosomal point mutation
IHIT48350	09/2015	ETEC-like	2944	-	-	*parE* I355T
**Farm 3 (*n* = 16)**	10/2014 – 04/2019	ETEC/STEC (37.5%), ETEC (18.8%), ETEC-like (18.8%), EDEC (12.5%), STEC (12.5%)	ST86 (43.8), ST1 (18.8%), ST10 (12.5%), ST90 (6.3%), ST100 (6.3%), ST118 (6.3%), ST162 (6.3%)	*mcr-1.1* (50%), *mcr-4.8* (6.3%)	*dfrA1* (75%), *aph(3``)-Ib* (68.8%), *aph(6)-Id* (68.8%), *bla*TEM-1B (68.8%), *sul1* (68.8%), *sul2* (68.8%), *aadA1* (62.5%), *tet(A)* (62.5%), *tet(B)* (56.3%), *sul3* (50%), *aadA13* (43.8%), *catA1* (43.8%), *aph(3`)-Ia* (37.5%), *aac(3)-IV* (18.8%), *aph(4)-Ia* (18.8%), *floR-like* (18.8%), *bla*TEM-1A (12.5%), *aadA5* (6.3%), *ant(3``)-Ia* (6.3%), *catB3* (6.3%), *dfrA14* (6.3%) *mph(B)* (6.3%), *mph(G)* (6.3%), *mef(C)* (6.3%)	*gyrA* S83L (50%), *parE* I355T (43.8%), *ampC*-prom 42C>T (18.8%)
IHIT34769	06/2017	EDEC	1	*mcr-1.1*	-	-
IHIT48353	06/2017	STEC	1	*mcr-1.1*	*aadA5, aph(3``)-Ib, aph(6)-Id, bla*TEM-1B*, catB3, dfrA1, dfrA14, sul1, sul2, tet(A)*	-
IHIT47062	10/2017	EDEC	1	-	-	-
IHIT48352	12/2016	STEC	10	*mcr-1.1*	*aph(3``)-Ib, aph(6)-Id, bla*TEM-1B*, dfrA1, sul1, sul2, tet(B)*	-
IHIT39537	04/2019	ETEC-like	10	*mcr-4.8*	*bla*TEM-1A	-
IHIT47044	10/2014	ETEC/STEC	86	*mcr-1.1*	*aac(3)-IV, aadA1, aadA13, aph(3`)-Ia, aph(3``)-Ib, aph(4)-Ia, aph(6)-Id, bla*TEM-1B*, catA1, dfrA1, floR-like, sul1, sul2, sul3, tet(A), tet(B)*	*gyrA* S83L, *parE* I355T
IHIT47045	10/2014	ETEC/STEC	86	-	*aac(3)-IV, aadA1, aadA13, aph(3`)-Ia, aph(3``)-Ib, aph(4)-Ia, aph(6)-Id, bla*TEM-1B*, catA1, dfrA1, floR-like, sul1, sul2, sul3, tet(A), tet(B)*	*gyrA* S83L, *parE* I355T
IHIT47046	06/2015	ETEC/STEC	86	*mcr-1.1*	*aac(3)-IV, aadA1, aadA13, aph(3`)-Ia, aph(3``)-Ib, aph(4)-Ia, aph(6)-Id, bla*TEM-1B*, catA1, dfrA1, floR-like, sul1, sul2, sul3, tet(A), tet(B)*	*gyrA* S83L, *parE* I355T
IHIT47048	06/2015	ETEC/STEC	86	-	*aadA1, aadA13, aph(3`)-Ia, aph(3``)-Ib, aph(6)-Id, bla*TEM-1B*, catA1, dfrA1, sul1, sul2, sul3, tet(A), tet(B)*	*gyrA* S83L, *parE* I355T
IHIT47056	06/2016	ETEC/STEC	86	*mcr-1.1*	*aadA1, aadA13, ant(3``)-Ia, aph(3`)-Ia, aph(3``)-Ib, aph(6)-Id, bla*TEM-1B*, catA1, dfrA1, sul1, sul2, sul3, tet(A), tet(B)*	*gyrA* S83L, *parE* I355T, *ampC*-prom 42C>T
IHIT47057	11/2016	ETEC	86	-	*aadA1, aadA13, aph(3`)-Ia, aph(3``)-Ib, aph(6)-Id, bla*TEM-1B*, catA1, dfrA1, sul1, sul2, sul3, tet(A), tet(B)*	*gyrA* S83L, *parE* I355T, *ampC*-prom 42C>T
IHIT34315	04/2017	ETEC/STEC	86	*mcr-1.1*	*aadA1, aadA13, aph(3``)-Ib, aph(6)-Id, bla*TEM-1B*, catA1, dfrA1, sul1, sul2, sul3, tet(A), tet(B)*	*gyrA* S83L, *parE* I355T, *ampC*-prom 42C>T
IHIT47060	02/2017	ETEC	90	-	*aadA1, aph(3``)-Ib, aph(6)-Id, bla*TEM-1B*, dfrA1, mph(B), mph(G), mef(C), sul1, sul2, tet(B)*	-
IHIT47047	06/2015	ETEC	100	*mcr-1.1*	*aadA1, bla*TEM-1A*, dfrA1, sul3, tet(A)*	-
IHIT47065	07/2018	ETEC-like	118	-	*aph(3``)-Ib, aph(6)-Id, aadA1, bla*TEM-1B*, dfrA1, sul1, sul2, tet(A)*	-

**Strain ID**	**Date of isolation**	**Pathotype***	**ST**	***mcr* gene**	**Further resistance genes**	**Chromosomal point mutation**
IHIT47072	03/2019	ETEC-like	162	-	-	*gyrA* S83L

* Pathotypes: AdhF-*Ec*, positive for at least one adhesive fimbriae gene (*faeG, fanA, fasA, fedA, fimF41a*); AEEC, positive for *eae*; EDEC, positive for *fedA* and *stx2*; ETEC, positive for at least one adhesive fimbriae gene (*faeG, fanA, fasA, fedA, fimF41a*) and at least one enterotoxin gene (*eltB-Ip, estap, estb*); ETEC-like, positive for at least one enterotoxin gene (*eltB-Ip, estap, estb*); ETEC/STEC, positive for at least one adhesive fimbriae gene (*faeG, fanA, fasA, fedA, fimF41a*) and at least one enterotoxin gene (*eltB-Ip, estap, estb*) and *stx2*; STEC, positive for *stx2*.
